# Opening large-conductance potassium channels selectively induced cell death of triple-negative breast cancer

**DOI:** 10.1186/s12885-020-07071-1

**Published:** 2020-06-26

**Authors:** Gina Sizemore, Sarah McLaughlin, Mackenzie Newman, Kathleen Brundage, Amanda Ammer, Karen Martin, Elena Pugacheva, James Coad, Malcolm D. Mattes, Han-Gang Yu

**Affiliations:** 1 Clinical and Translational Sciences Institute, West Virginia University, Morgantown, USA; 2 Animal Models & Imaging Facility, Cancer Institute, West Virginia University, Morgantown, USA; 3 Department of Physiology & Pharmacology, West Virginia University, Morgantown, WV 26506 USA; 4 Department of Microbiology and Cell Biology, Flow Cytometry Facility, West Virginia University, Morgantown, USA; 5 Department of Biochemistry, Cancer Institute, West Virginia University, Morgantown, USA; 6 Department of Pathology, West Virginia University, Morgantown, USA; 7 Department of Radiation Oncology, Cancer Institute, West Virginia University, Morgantown, USA

**Keywords:** Potassium channels, Hyperpolarization, Triple-negative breast cancer

## Abstract

**Background:**

Unlike other breast cancer subtypes that may be treated with a variety of hormonal or targeted therapies, there is a need to identify new, effective targets for triple-negative breast cancer (TNBC). It has recently been recognized that membrane potential is depolarized in breast cancer cells. The primary objective of the study is to explore whether hyperpolarization induced by opening potassium channels may provide a new strategy for treatment of TNBC.

**Methods:**

Breast cancer datasets in cBioPortal for cancer genomics was used to search for ion channel gene expression. Immunoblots and immunohistochemistry were used for protein expression in culture cells and in the patient tissues. Electrophysiological patch clamp techniques were used to study properties of BK channels in culture cells. Flow cytometry and fluorescence microscope were used for cell viability and cell cycle studies. Ultrasound imaging was used to study xenograft in female NSG mice.

**Results:**

In large datasets of breast cancer patients, we identified a gene, KCNMA1 (encoding for a voltage- and calcium-dependent large-conductance potassium channel, called BK channel), overexpressed in triple-negative breast cancer patients. Although overexpressed, 99% of channels are closed in TNBC cells. Opening BK channels hyperpolarized membrane potential, which induced cell cycle arrest in G2 phase and apoptosis via caspase-3 activation. In a TNBC cell induced xenograft model, treatment with a BK channel opener significantly slowed tumor growth without cardiac toxicity.

**Conclusions:**

Our results support the idea that hyperpolarization induced by opening BK channel in TNBC cells can become a new strategy for development of a targeted therapy in TNBC.

## Background

The main molecular subtypes of breast cancer are termed Luminal A (ER+/HER2-), Luminal B (ER+/HER2+/−, higher histological grade, more aggressive than Luminal A), HER2-enriched (ER−/HER2+), and triple-negative (ER−/PR−/HER2-) [1]. Recent gene expression studies further identified five “transcriptional subtypes” of breast cancer: basal-like, HER2-enriched, luminal A, luminal B, and normal-like (now thought not to originate from breast cancer) [2]. Up to 70% of triple-negative breast cancer (TNBC) have the basal-like gene expression signatures, however, a large number of basal-like tumors express ER, PR or HER2 [3]. Further studies on molecular signatures, genetics, and genomics have led to the identification of four TNBC subtypes (basal-like 1, basal-like 2, mesenchymal, and luminal androgen receptor) [4, 5]. These studies have revealed the complexity of breast tumors and generated many new hypotheses for potential therapeutic targets for treatment of TNBC.

TNBC is one of the subtypes of breast cancer with an earlier onset, more aggressive metastasis, and lacks the therapies available to ER+, PR+, and HER2+ breast cancers [3]. Five-year breast cancer survival is significantly reduced with diagnosis of TNBC [6], due largely to relatively ineffective therapeutic options [7]. There is therefore a need to identify new, effective targets for TNBC.

All cells maintain a polarized membrane potential (Em), more negative inside than outside the cell membrane. Em is essential to the development of action potentials in excitable cells such as neurons and cardiac myocytes. However, accumulating evidence has also demonstrated variability of Em in non-excitable epithelial cells and cancer cells as well [8]. Alterations in Em (depolarization – i.e. Em becoming more positive, hyperpolarization – Em becoming more negative) are now recognized to play a crucial role in controlling the cell cycles [9, 10].

Using a traditional microelectrode technique, Em was reported to be -13 mV in breast cancer biopsy specimens from nine women with infiltrating ductal carcinoma, independent of ER or PR presence [11]. For comparison, normal human breast epithelial cell Em is near -60 mV [11]. Thus, Em is depolarized in breast cancer compared to normal breast cells. Using whole-cell patch clamp, we found more positive Em in TNBC MDA-MB-231 cells (− 39.5 mV) than in normal breast cells (− 66.9 mV) [12].

KCNMA1 gene encodes the pore-forming alpha subunit of a voltage- and calcium-gated large-conductance potassium channel, called BK (also known as Slo1, Maxi-K, or KCa1.1) channel [13, 14]. Previous studies have suggested a contradictory role of KCNMA1 in breast cancer proliferation, invasion, and metastasis. Blockade of BK channels can slow proliferation and invasion of breast cancer cells [15, 16]. In contrast, studies with anti-tumor compounds revealed anti-tumor action as an important result with activation of BK channels in metastatic breast cancer cells [17].

BK channels have to open (activate) to exert their function – hyperpolarizing Em by loss of intracellular potassium ions. Therefore, high expression of KCNMA1 does not necessarily guarantee high activity because closed channels do not have activity and cannot hyperpolarize the cell. Similarly, low expression of KCNMA1 expression levels can have a significant impact if all channels are open. Therefore, the strategy to inhibit or activate BK channels can only be decided after we determine if the channels are open or closed at the Em of TNBC cells.

In this work, we present evidence for overexpression of KCNMA1 in TNBC from a large dataset. Second, we verify a significant increase in protein expression of BK channels in TNBC cell lines and primary TNBC tissues. Third, we provide an answer to an intriguing question regarding how breast cancer cells remain depolarized while overexpressing a hyperpolarizing ion channel, which should make cell membrane potential more negative. Fourth, we demonstrated that opening BK channels hyperpolarizes Em and induces apoptotic death of TNBC cells via activated caspase-3. Fifth, we show that BK channel openers can slow tumor growth in an MDA-MB-231 xenograft model in female NSG mice to validate the main in vitro finding. Finally, we show that this new approach of using BK channel openers for selective induction of death in TNBC does not impact healthy breast tissue and cardiac function.

## Methods

### KCNMA1 gene expression in the Cancer genomic atlas (TCGA) database

KCNMA1 gene expression patterns in primary breast cancer database were analyzed from The Cancer Genome Atlas (TCGA) [18] via cBioPortal (http://cbioportal.org) [19]. Gene expression levels from RNA-sequencing data was illustrated in log2-fold of fragments per kilobase of transcript per million (FPKM) [20].

### Cell culture and plasmid transfection

Human breast adenocarcinoma cells (ER+ MCF7, triple-negative MDA-MB-231/Luc and SUM159) were grown in Dulbecco’s modified Eagle’s medium (DMEM, Invitrogen), whereas triple-negative HCC1143 cells were grown in RPMI (Invitrogen), supplemented with 10% fetal bovine serum, 1X Pen/Strep (Gbico 15140). Normal human mammary epithelial cells (MCF10A) were grown in mammary epithelial cell basal medium (MEC), supplemented with MEC growth kit (ATCC). Rat h9c2 cardiac myocytes were purchased from ATCC (CRL-1446), cultured in DMEM. All human cell lines were obtained five years ago from American Type Cell Collection (ATCC) in which authentication was performed using immunoblots. ATCC catalog numbers are CRL-10317 for MCF10A, HTB-22 for MCF7, HTB-26 for MDA-MB-231, and CRL-2321 for HCC1143. SUM159 was a gift from Dr. Elena Pugacheva (co-author) that was authenticated using immunoblots. We did not reauthenticate MCF10A, MCF7, MDA-MB-231, and HCC1143. All cell lines used in this study were tested negative for mycoplasma contamination using Mycoplama Detection Kit (InvivoGen, PlasmoTest, Cat#: rep-pt1).

Cells with 50–70% confluence in 6-well plates were used for transient plasmid (1-2 μg) transfection using Lipofectamine3000 transfection reagent (Invitrogen). Human wild type (hSlo1 WT) and A313D mutant (hSlo1-A313D) channel cDNA in pcDNA3 mammalian expression vector were co-transfected with GFP for verification of expression.

### Patch clamp studies in MDA-MB-231

Details in whole-cell or perforated (or permeabilized) patch clamp studies in isolated cells have been previously reported [21, 22]. Briefly, the cells grown on coverslips were placed in a lucite bath with the temperature maintained at 35 °C - 37 °C. Em and voltage-gated potassium currents were recorded using the whole cell patch clamp technique with an Axopatch-700B amplifier. Em was measured with DMEM or Tyrode solution and pipette solution. DMEM contained physiological ion concentration (in mM): 150Na^+^, 5 K^+^, 2.0Ca^2+^ (pH = 7.4). Tyrode solution contains (mM): NaCl 140, KCl 5.4, CaCl_2_ 1.8, MgCl_2_ 1, Glucose 5.5, Hepes 5, pH 7.4 adjusted by NaOH. The pipette solution contained (in mM): 85 KCl, 40 K-Aspartate, 0.1CaCl, 10 HEPES, pH was adjusted to 7.2 by KOH. The pipettes had a resistance of 2–5 MΩ when filled with pipette solution. For perforated patch, amphotericin - B was added to the pipette solution to a final concentration of 240 μg/ml. The whole-cell/perforated patch clamp data were acquired by CLAMPEX and analyzed by CLAMPFIT (pClamp 9, Axon/Molecular Device).

### Live cell imaging

Live cell imaging experiments were performed using a Zeiss Axio Observer A1 inverted microscope with fluorescence. Images were acquired and analyzed using AxioVision (version 4.6). We used ethidium homodimer-1 (EthD-1, Invitrogen, 0.2–0.5 μl of 2 mM stock to 1 ml culture of cells in 6-well plates) to label dead cells (Fig. 4; Supplemental Figs. 3–7). Ethidium homodimer assay was utilized to detect dead cells. Ethidium homodimer is impermeable to the membrane of a living cell. However, when the cell dies the ethidium homodimer fluorescent dye is able to bind to the DNA, emitting bright red fluorescent signals. Cell count was performed using ImageJ (NIH).

### Western blotting

Cells were harvested using Radioimmunoprecipitation Assay (RIPA) buffer with 1% protease inhibitor cocktail (Sigma). We then sonicated lysates on ice and centrifuged at 12,000×g for 10 min at 4 °C. Tumor tissue was homogenized in RIPA buffer with 1% protease inhibitor cocktail (Sigma), then centrifuged at 12,000×g for 10 min at 4 °C. Supernatant was isolated from debris pellet.

Protein concentration was measured using Bicinchoninic acid assay (BCA) (Thermo Fisher). Once protein concentrations were normalized across samples, they were then heated for 12 min at 90 °C. Samples were loaded into NuPage 4–12% bis-tris gels (Invitrogen) with MOPS running buffer at 70 V for 100 min, then transferred to 0.2 μm pore PVDF membrane (Thermo Scientific) at 30 V for one hour in cold room. Next, blots were blocked in Licor blocking buffer for one hour, and incubated for 12 h at 4 degrees with primary antibody for either anti-KCNMA1 for epitope 199–213 (Alomone cat# APC-151), anti-caspase-3 (Cell Signaling Technology), or anti-Slo1 for c-terminus segment 9–10 (Millipore) at 1:500 dilution. The membrane was then washed 3 times for 15 min with tris-buffered saline containing 0.1% tween-20 (TBS-T). A secondary antibody, IR 800 CW from Licor (1:20,000 dilution) was incubated with the membrane at room temperature for one hour. After three 10-min washes with TBS-T, blots were imaged using a Licor Odyssey CLx and image studio software. If residual background signal was observed, additional washes of 5 to 10 min with TBS-T were completed and the membrane was re-imaged. Beta-actin primary antibody (Proteintech) and IRDye 680RD secondary antibody (Licor) were used as a loading control.

### Immunohistochemistry of patient breast samples

Experiments involving patient breast samples were approved by West Virginia University Institutional Review Board. Formalin-fixed paraffin-embedded (FFPE) breast tumor tissue from patients was processed according to vendor’s manual instruction (Biocare) and following a verified protocol in the Pathology Laboratory of Translational Medicine at WVU. Briefly, 3 μm sections were deparaffinized on slides, quenched with hydrogen peroxide, and incubated in BK channel antibody (Sigma-Aldrich, HPA054648) Sigma at 4 °C for 4 min. Horseradish peroxidase-containing secondary antibody (UMap anti-RB, Roche, Diagnostic, Cupertino, CA) was then added for 8 min and developed using Biocare DAB (brown color). Hematoxylin was used as a counterstain (blue color).

Automated formalin-fixed, paraffin embedded immunohistochemical staining, to evaluate tumor antigen expression profiles, is available via the Ventana DISCOVERY automated IHC Platform. Breast tumor IHC slides stained with BK channel antibody were examined under an Olympus VS-120 slide scanner with a 10X Plan S Apo/0.40 NA objective, equipped with a color camera (Pike 505C VS50). The images were analyzed using OlyVIA (Olympus) and ImageJ (NIH). Percentage of area staining was used to quantify the protein expression levels in IHC slides.

### Breast tumor induction in NOD scid gamma (NSG) mice

Female NSG-immunodeficient mice of 4–6 weeks old were purchased from the Jackson Laboratory. Experimental procedures and housing of the animals were approved by the Institutional Animal Care and Use Committee. Animal were housed in a fully state-of-the-art facility that includes large specific pathogen free rooms, husbandry conditions (breeding program, light/dark cycle, temperature control, quality water, clean cages access to food and water), and welfare-related policies related to tumor studies (e.g., tumor burden policy).

Following power analysis, a total of 16 female mice was used, 8 for treatment, 8 for controls. For mammary pad injections, pathogen-free luciferase-expressing human breast adenocarcinoma cells (MDA-MB-231/Luc, 1-2 × 10^6^ cells/animal) were injected into the fourth inguinal mammary gland of 6- to 8-week-old mice. Primary tumors had formed typically two weeks following cell injection. Tumor size was monitor by imaging twice a week. After experiment, mice were euthanized by isoflurane overdose (5% to effect or an overdose 100 mg/kg of sodium pentobarbital), a procedure approved by our IACUC.

### Ultrasound imaging of xenograft tumor in NSG mice

Details for ultrasound imaging of xenograft tumor in NSG mice have been previously reported [21]. Briefly, animals were anesthetized by exposure to 1–3% isoflurane during imaging. Imaging was performed weekly over the course of each experiment, typically for 4–6 weeks. Tumor volume was imaged by ultrasound imaging (USI) with Vevo2100 Micro-Ultrasound System. A 40 or 50 mHz transducer was used, depending on the tumor volume. A 3-dimensional (3D) image was acquired with scanning distance of 0.071 mm between images. Vevo software then integrated the images into a reconstructed 3D tumor from which the tumor volume was obtained.

### BK channel openers

BMS-191011 (Tocris) and NS11021 (Tocris) were prepared in 10 mM DMSO stock. The working concentrations of 10-50 μM contained 1-5 μl of DMSO in 1 mL DMEM medium, resulting in 0.1–0.5% of DMSO, which did not affect TNBC cells (Fig. S7). For testing in vivo effects of MDA-MD-231, 3 μl of 10 mM (equivalent to 186.82 ng) stock was added to 1 mL PBS, 50 μl was administrated directly into the xenograft grown in mouse via during day time in the animal imaging facility. For testing adverse effects of the drug, tail-vein injection was used.

### Scratch (or “wound healing”) assay

MDA-MB-231 cells were incubated in a 24-well plate. After reaching confluence, the scratch (or “wound”) was created using a sterile 200-μl pipette tip, defined by the space within two red lines (upper left), filled (or “healed”) by migration of cells (upper right). Curved red line indicates the marker (shadowed area) used to identify the location of the scratch. Each petri dish reached 70% confluence before performing the assay. The difference between the control (untreated) and treated cell growth was visually demonstrated by less than 10 live cells in the BMS-191011 treated “wound” region (Supplemental Fig. 10D), and a complete repopulation in the control (Supplemental Fig. 10B).

### Caspase-3/7 green fluorescence dye

CellEvent Caspase-3/7 Green Detection Reagent was obtained from Thermo Fisher (Cat#: C10723). Working solution of 1 μM was used in cell culture. Fluorescence microscopy was used to acquire images. Green fluorescence was detected only apoptotic cells.

### Cell cycle analysis using flow cytometry

Cells were grown to 60–80% confluency in DMEM before drug treatment. Cells were either treated with BMS-191011 in DMSO, DMSO alone, or no treatment. After 24–48 h, cells were washed with PBS and incubated with 0.25% trypsin with EDTA (Invitrogen) for 5 min at 37 °C. After combining the resultant solution with 10 mL PBS in 15 mL tubes, cells were pelleted at 1000 rpm for 6 min at 4 °C. Cell pellets were resuspended in 200 μL PBS then added with swirling to 2 mL ice cold 70% ethanol. These single-cell suspensions were kept at 4 °C until further processing.

For flow cytometry, cells were re-pelleted at 1000 rpm for 6 min before decanting ethanol. After resuspension in 2 mL PBS and incubation for 1 min at room temperature, cells were pelleted and resuspended in 100 μL of 0.2% Tween-20 in PBS at room temperature then incubated at 37 °C for 15 min. Next, 100 μL of PBS containing 2% fetal bovine serum and 0.1% sodium azide was added and cells were pelleted. The solution was then decanted, 10 μL of RNase A (DNase- and protease-free; Thermo Scientific EN0531) in PBS (180 μg/mL) was added and allowed to sit for 15 min at room temperature, and 20 μL of propidium iodide (PI) in PBS (50 μg/mL) was added for 15 min. The resultant solution was brought to a volume of 300-500 μL using PBS before data collection.

Samples were analyzed on BD LSRFortessa using BD FACS Diva version 8.0 software. A minimum of 20,000 cells were collected for each sample. Data analysis was done using FCS Express 6.0 flow cytometry software (De Novo Software, Los Angeles CA). Cell cycle fit algorithm was selected using the lowest relative chi square value and BAD value < 20%. The sub-G1 peak in DNA profile plots was gated out to focus on altered distribution of G1/S/G2.

### Statistical analysis

Data were shown as mean ± standard deviation (SD) in the text. Bar figures were presented as mean ± SD using GraphPad (Prism). Student’s t-test and two-way ANOVA (for more than two groups) were used for statistical analysis. *P* < 0.05 was considered as statistically significant. Details in statistical analysis, such as F values and degree of freedom (DF) when using ANOVA and t values when using t-test, are included in the figure legends.

## Results

### KCNMA1 gene and protein expression in breast cancer patients

**Fig. 1:**
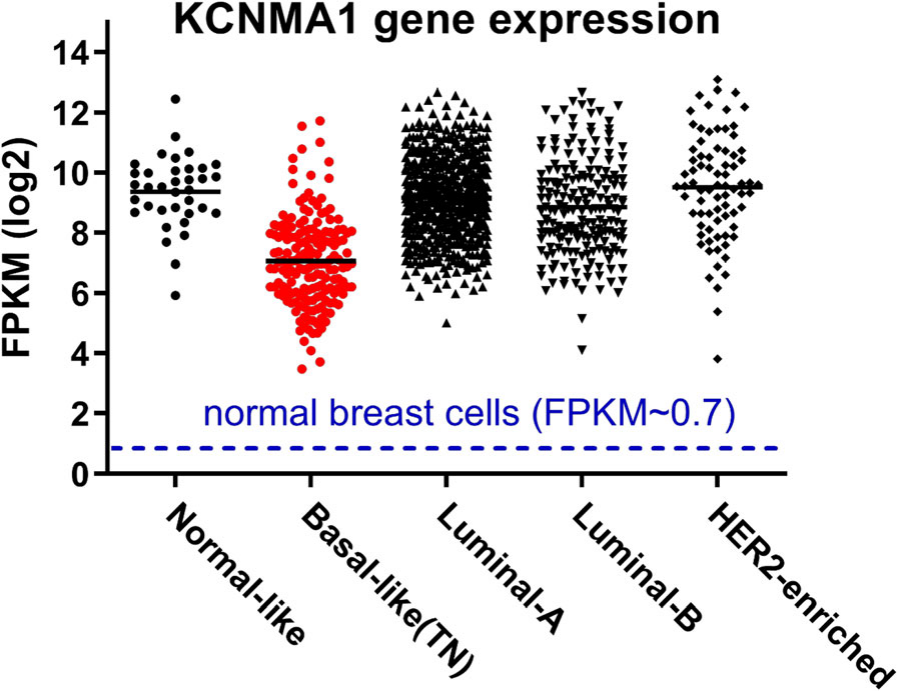
KCNMA1 gene expression in five molecular subtypes of breast cancer. Each dot represents one patient. Gene expression levels are presented as FPKM in log2 expression. Red dots are basal-like triple-negative (TN) breast cancer patients. Blue dotted line indicates KCNMA1 gene expression in normal breast cells. FPKM: Fragments Per Kilobase of transcript per Million

We used gene expression data (RNA-seq) from 981 breast cancer samples (TCGA Cell 2015 [23] and TCGA Provisional). In all five subtypes BK channel KCNMA1 gene expression levels are dramatically upregulated, comparison to normal breast cells (FPKM ~ 0.7) (Fig. 1). TNBC patients are represented by red dots.

Using transcriptomics and a targeted proteomics approach, the gene-specific correlation of mRNA levels and protein copy number has been well established in human cells and tissues [24]. Previous studies have demonstrated that BK channel alpha subunit protein (main subunit forming the pore of the channel) is abundantly expressed in MDA-MB-231 cells, weakly expressed in MCF7, and nearly undetectable in normal breast epithelial cells MCF10A [15]. Therefore, we set out to investigate the protein expression of BK channels in TNBC patients’ tissues.

**Fig. 2:**
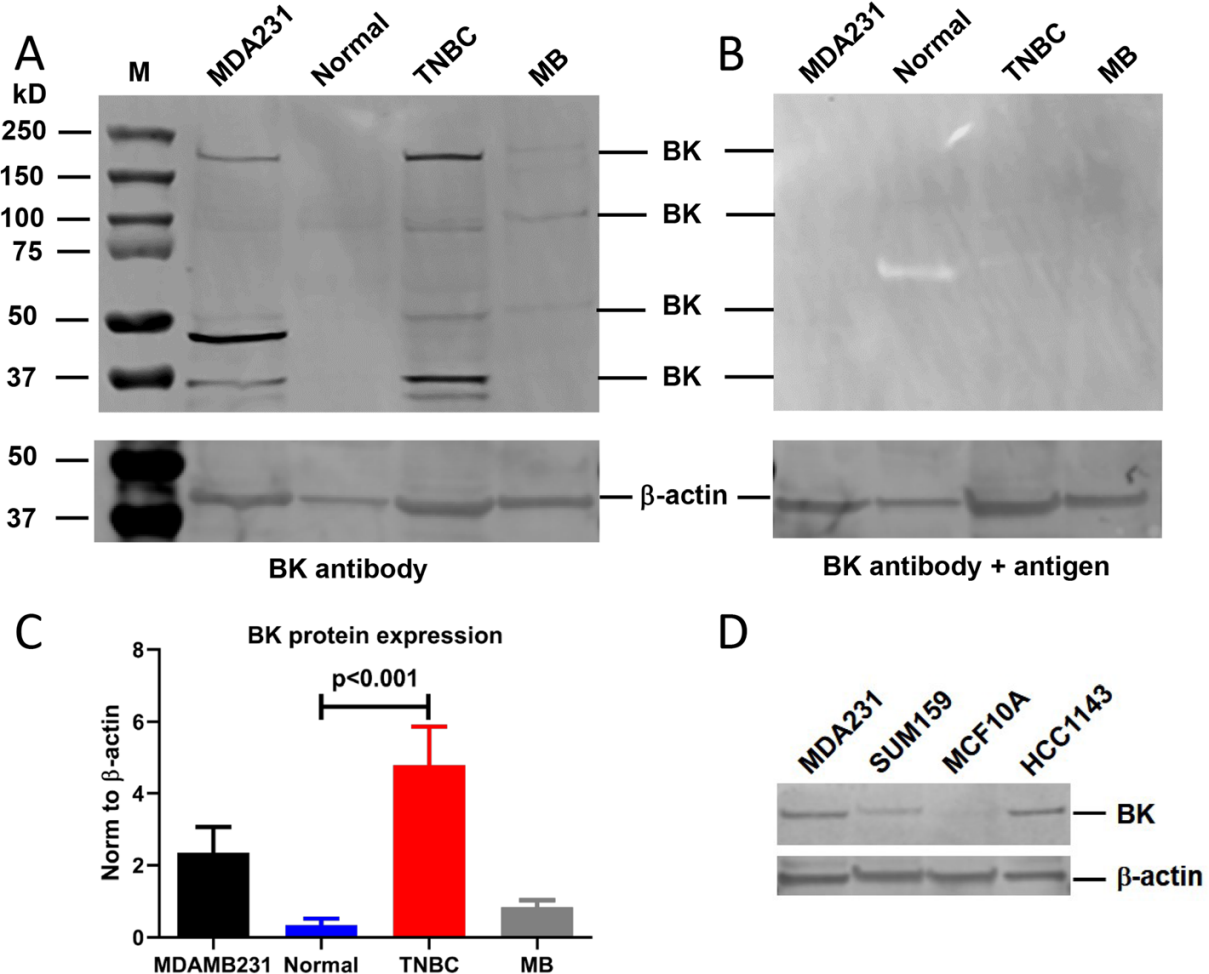
Protein expression of BK channel α-subunit (forming the functional channel) in TNBC cells and patient tissues. **a** Western blots with antibody, M: marker, MDA231: MDA-MB-231 cells (cell positive control), Normal: normal primary breast tissue, TNBC: TNBC patient tissue, MB: mouse brain (tissue positive control). β-actin was used as a loading control. **b** Western blots with antibody and antigen, confirming signals detected by the antibody in (**a**). Figure 2a and b were prepared by cropping the full WB shown in Supplemental Fig. 15 by removing the white spaces above and below the signals of interest. **c** Quantitative expression levels of BK channel proteins normalized to β-actin (*n* = 4–6). Unpaired t test was performed. Two-tailed *p* = 0.0002, t = 0.8172. **d** Western blots using a BK channel protein antibody in MDA231, SUM159, MCF10A, and HCC1143, β-actin as loading controls. Figure 2d was prepared by cropping the full WB in Supplemental Fig. 16 by removing the white spaces above and below the signals of interest

Figure 2 shows a representative figure for protein expression of BK channel alpha subunit in primary TNBC tissue using an antibody that targets an epitope in the 1st extracellular loop of transmembrane domains 1 and 2 (corresponding to amino acid residues 199–213 of rat KCNMA1 (Alomone Labs). MDA-MB-231 (MDA231 in the figure) was used as a positive control. Mouse brain (MB) known to express BK channels [25, 26] was used as an additional positive control (stronger signals in a more sensitive fluorescence image of Western blot is provided in **Supplemental Fig.** **1**). In addition to the glycosylated channel protein (around 200kD), there exist smaller fragments recognized by the antibody that are likely the proteolyzed C-terminals of the channel protein reported in previous studies [27]. The interpretation of the results was confirmed by incubation of the antigen (**2B**) that showed disappearance of the signals in **2A**. After total expression signals being normalized to β-actin, BK channel protein expression levels are nearly 14-fold higher in primary TNBC than in normal human breast tissue (Normal: 0.345 ± 0.177; TNBC: 4.793 ± 1.074, *n* = 4–6, *p* < 0.001) (**2C**). Figure 2**d** shows that BK channel proteins are also abundantly expressed in different types of TNBC cells (SUM159, HCC1143), but barely detectable in MCF10A normal breast cells.

To confirm the increased protein expression levels of BK channels in TNBC patients, we performed IHC experiments in seven TNBC tissue and three normal breast tissue samples. We used a BK channel antibody that had been successfully applied in identifying KCNMA1 channel protein expression in the Human Protein Atlas (Sigma-Aldrich, HPA054648). **Supplemental Fig.** **2** shows a normal breast tissue (**A**) and a TNBC tissue (**B**). The averaged percentage of protein expression area is shown in **(C)**. BK channel protein levels were increased by ~ 9-fold in TNBC than in normal breast tissue (TNBC = 3.56 ± 1.33, *n* = 7; Normal = 0.41 ± 0.10, *n* = 3; *p* < 0.01).

Figure 1 raised several questions: 1) Why are depolarized TNBC cells overexpressing a hyperpolarizing BK channel? 2) Are these overexpressed BK channels activated (open)? These questions led us to the hypothesis that these overexpressed BK channels are not activated. If this hypothesis is correct, then opening these channels can be exploited as a novel strategy for targeted therapy in treatment of TNBC.

For ion channels whose activity is dependent on Em, gene/protein expression levels are not intrinsically correlated with channel activity. At the resting Em of the cell, ion channels are active when they are open, inactive when they are closed. We therefore set up to investigate biophysical properties of BK channels in TNBC cells.

### Voltage-dependent activation of BK channels in TNBC cells

Previously, we showed that the resting Em, which is within the physiological voltage range, in MDA-MB-231 cells is depolarized compared to normal mammary epithelial cells (HMEC) (Em_MDA-MB-231: about -40 mV, Em_HMEC: about -67 mV) [12]. To investigate whether BK channels in MDA-MB-231 are open at -40 mV, we studied biophysical properties of BK channels in MDA-MB-231 cells using whole-cell and perforated patch clamp techniques.

**Fig. 3:**
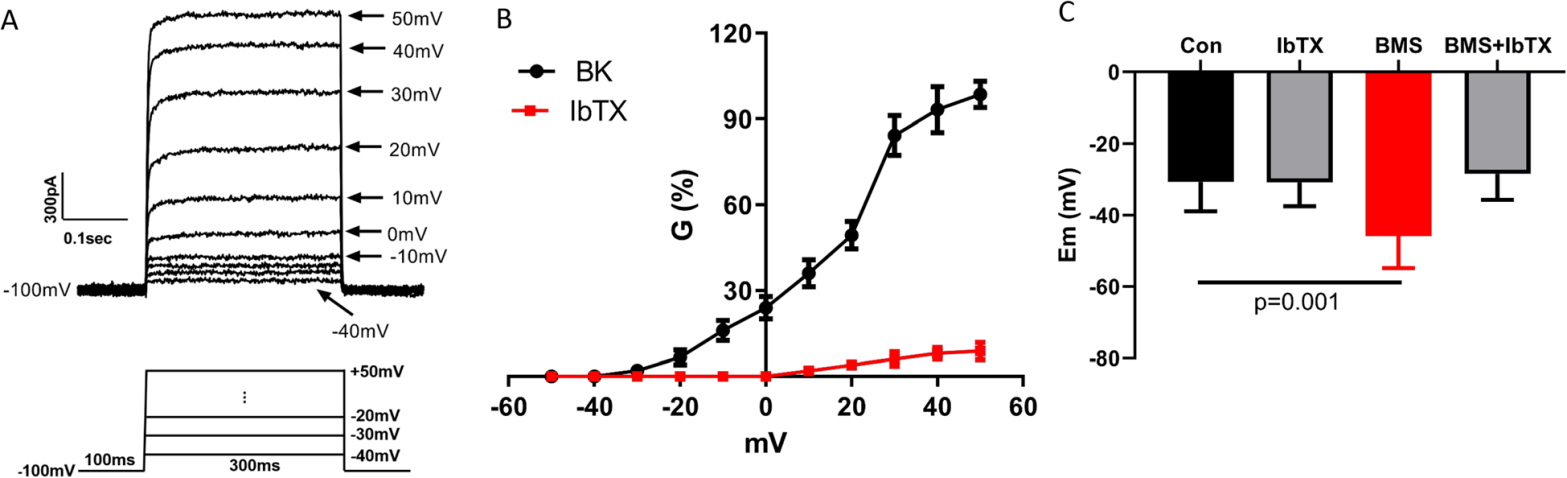
Biophysical properties of BK channels in MDA-MB-231 cells. **a** BK channel currents elicited by depolarizing pulses ranging from -40 mV to + 50 mV in 10 mV increment, pulse protocol is shown below. **b** BK channel conductance, G, − voltage relationship in the absence (black) and presence (red) of 0.1 μM IbTX (*n* = 8). **c** Effects of BMS-191011 and IbTX on membrane potential (Em) in MDA-MB-231

Figure 3a shows the typical BK channel currents activated by the depolarizing pulse protocol (below **3A**). The currents were confirmed to be generated from BK channels by iberiotoxin (IbTX, 100 nM) (known as a potent specific blocker of BK channel (with IC_50_ of 250pM) since it does not affect other ion channels [28]). Figure 3b shows the average voltage-dependent activation curve of BK channel in eight (8) MDA-MB-231 cells. At -40 mV (resting Em in MDA-MB-231), only 1% of BK channels are open.

### BK channel opener hyperpolarizes Em in MDA-MB-231 cells

Opening large conductance (>200pS) [29] BK channels causes loss of intracellular K^+^ ions to the outside of the cell, leading to membrane hyperpolarization. In MDA-MB-231 cells, we used a potent selective BK channel opener, BMS-191011 (1 μM) [30] to study how opening BK channels may affect cell Em. We found that BMS-191011 (1 μM) hyperpolarized Em by 15 mV (Em_con = − 30.71 ± 8.20 mV, Em_BMS = − 45.86 ± 8.95 mV, *n* = 8; *p* < 0.01) within 15 min of application (Fig. 3c). IbTX (100 nM) did not induce significant change in Em (Em_con = − 30.71 ± 8.20 mV, Em_IbTX = − 30.86 ± 6.64 mV, *n* = 8; *p* > 0.05), but completely reversed hyperpolarization induced by BMS-191011 (Em_con = − 30.71 ± 8.20 mV, Em_BMS + IbTX = − 28.43 ± 7.30 mV, *n* = 8; *p* > 0.05) (Fig. 3c). The results provided additional evidence that the majority of BK channels are closed at Em in MDA-MB-231 cells.

### BK channel opener induced death of human TNBC cells

**Fig. 4:**
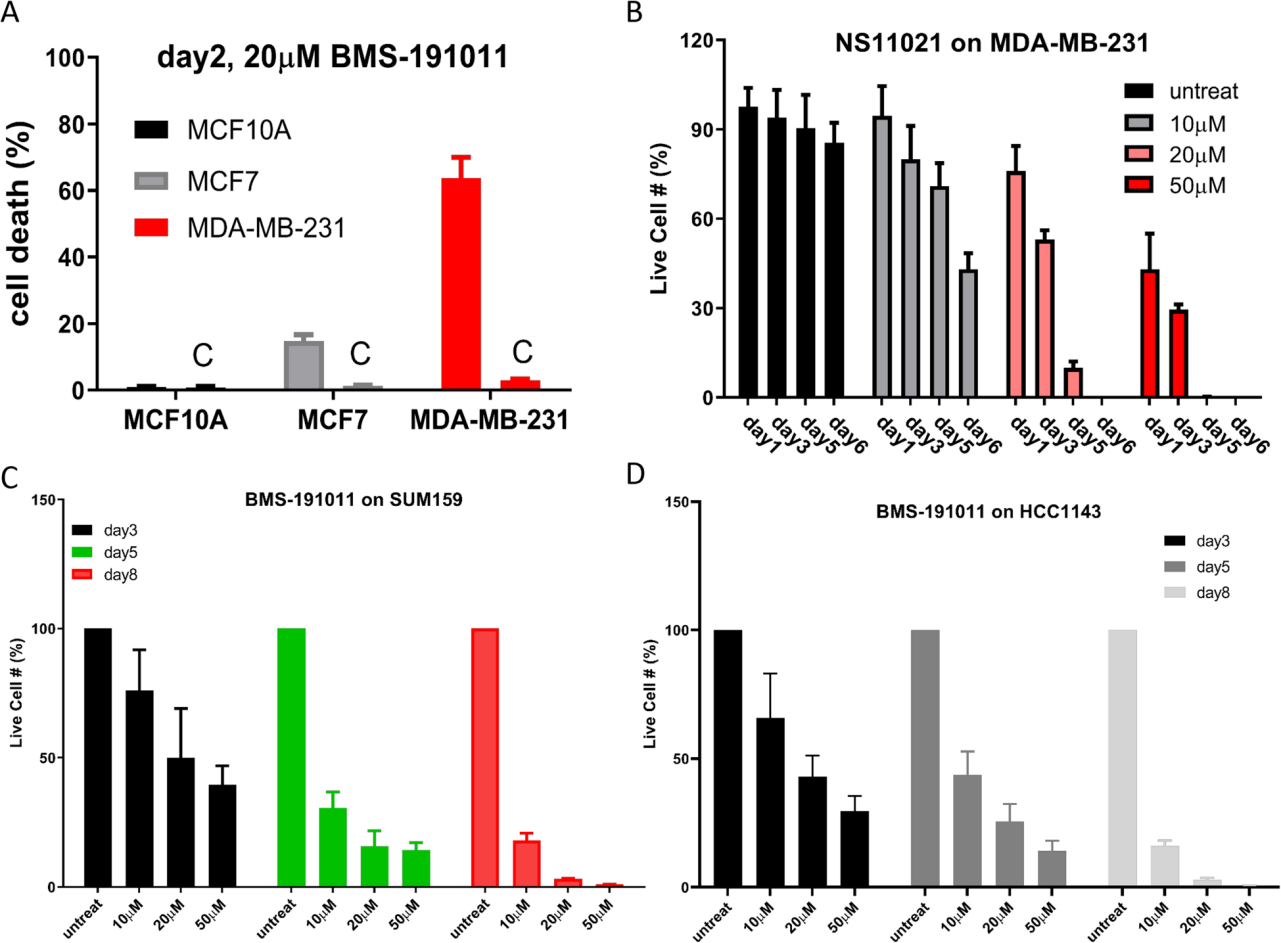
BK channel openers induced cell death in TNBC cell lines. **a** BMS-191011 (20 μM) induced cell death on normal breast cells (MCF10A, dark), ER+ breast cancer cells (MCF7, grey), and TNBC cells (MDA-MB-231, red) (*n* = 5). One-way ANOVA was performed, *p* < 0.0001, F = 65.38. **b** Time- and concentration-dependent effects of NS11021 on growth of MDA-MB-231 cells (*n* = 6). Two-way ANOVA was performed, *p* < 0.0001, F = 66.67, DF = 9. **c** Time-dependent effect of BMS-191011 (20 μM) on growth of SUM159 cells (*n* = 4). Two-way ANOVA was performed, *p* < 0.0001, F = 618.9, DF = 6. **d** Time-dependent effect of BMS-191011 (20 μM) on HCC1143 cells (*n* = 4). Two-way ANOVA was performed, *p* < 0.0001, F = 27.6, DF = 4

To test whether hyperpolarization by opening BK channels can induce death of TNBC cells, we studied effects of BK channel opener in TNBC cell lines. Treatment of BMS - 191,011 at 20 μM for two days did not affect normal breast MCF10A cells (**Supplemental Fig.****3****, left panels**), but dramatically induced cell death of MDA-MB-231 cells (**Supplemental Fig.****3****, middle panels**). BMS-191011 halted growth of MCF-7 cells but induced death of much fewer cells compared to MDA-MB-231 (**Supplemental Fig.****3****, right panels**). Figure 4a shows the percentages of dead/dying cells are 0.92 ± 0.29% (*n* = 5) for MCF10A, 14.76 ± 1.94% (*n* = 5) for MCF-7, and 63.82 ± 6.21% (*n* = 5) for MDA-MB-231, *p* < 0.0001 (One-way ANOVA).

To ensure that BMS-191011 induced cell death is via opening BK channels, we used another specific BK channel opener, NS11021, which has a different chemical structure [31]. Figure 4b shows time - and concentration – dependent effects of NS11021 on the growth of MDA-MB-231. At day 5, most cells treated with 20 μM NS11021 were dead (**Supplemental Fig.** **4**). For the same day, NS11021 induced cell death at different concentration is statistically significant compared to untreated group (*p* < 0.0001, *n* = 6).

To test whether BK channel opener mediated hyperpolarization-induced cell death is independent of TNBC subtypes, we studied effects of BMS-191011 on additional TNBC cell lines, SUM159 (Basal A, like MDA-MB-231) and HCC1143 (Basal B) [32]. BMS-191011 inhibited cell growth of SUM159 (**4C**) (and **Supplemental Fig.** **5**) and HCC1143 (**4D**) (and **Supplemental Fig.** **6**) cells in a similar way compared to that in MDA-MB-231. Additional controls were performed to rule out the potential side effects of DMSO on the cell growth of TNBC. **Supplemental Fig.** **7** shows an example for 0.5% DMSO (maximal volume used in drug treatment) that has no effect in cell death of HCC1143. We also tested that DMSO had no effects in cell growth of MDA-MB-231 and SUM159 cell lines.

Additionally, we tested whether a mutated BK channel that is permanently open (A313D), leading to hyperpolarization [33] may induce MDA-MB-231 cell death. **Supplemental Fig.** **8** shows that after 2 days of transfection, 59.4 ± 13.7% (*n* = 5) of MDA-MB-231 cells expressing A313D died, in comparison to 17.2 ± 5.3% (*n* = 5) of death in cells expressing wild-type (WT) channels or 18.8 ± 7.4% of death in cells expressing on GFP plasmid (*n* = 5, *p* < 0.003). After 4 days of transfection, all MDA-MB-231 cells expressing A313D were dead, whereas most cells expressing WT channels or GFP alone were alive.

### BK channel opener induced apoptosis and caspase-3 activation in TNBC

To understand the mechanism that mediates hyperpolarization - induced death in TNBC cells, we studied apoptosis, a well-studied programmed cell death mechanism. We performed time-lapse imaging experiment demonstrating that low concentration (1 μM) of BMS-191011 induced rapid cell shrinkage, a distinguished event unique to apoptosis [34], within 20-60 min (**Supplemental Fig.** **9**).

**Fig. 5:**
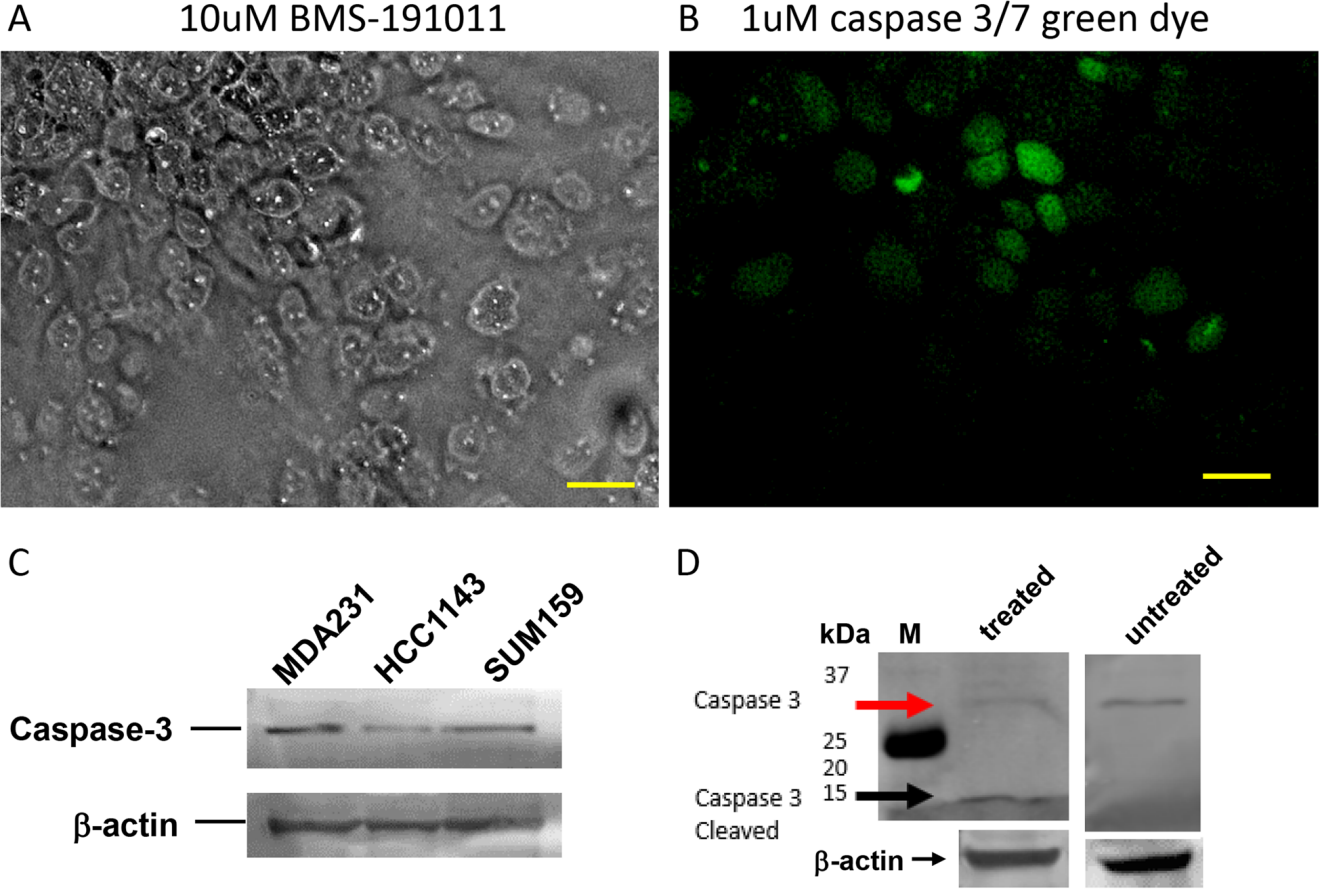
BMS-191011 induced activation of caspase-3 in MDA-MB-231. Using caspase-3/7 green dye, fluorescence was detected after incubation of 10 μM BMS-191011 for three days (**b**). Light image is shown in (**a**). Scale bar: 20 μm. Control cells are shown in the Supplemental Fig. 14. Using immunoblots, caspase-3 was detected in three TNBC cell lines (MDA-MB-231, HCC1143, SUM159) (**c**). Cleaved caspase-3 was detected after BMS-191011 treatment (**d**, M-marker, red arrow indicates pro-caspase 3, black arrow indicates cleaved active caspase-3). Figure 5c and d were prepared by cropping the full WB in Supplemental Figs. 17 and 18, respectively, by removing the white spaces above and below the signals of interest

We also studied effect of BK channel opener in caspase activation, an established mechanism and a strong indicator of apoptosis [35]. We first used a fluorescent caspase-3/7 green dye to test whether BK channel opener may induce caspase activation in MDA-MB-231. Figure 5 shows that after 3 days of 10 μM BMS-191011 treatment (**5A**), strong fluorescent signals (**5B**) were detected in MDA-MB-231. Furthermore, Fig. 5c shows the presence of pro-caspase-3 protein expression in three TNBC cell lines, MDA-MB-231, HCC1143, and SUM159. BMS-191011 (20 μM) treatment for two days induced cleaved caspase-3 expression in MDA-MB-231 (**5D**). These results suggested that BK channel opener can induce caspase-3 activation in MDA-MB-231 cells. Thus, hyperpolarization can induce activation of caspase-3 and apoptosis in TNBC cells.

### BK channel opener prevented migration of MDA-MB-231

Majority of breast cancer patients die due to tumor metastasis and one critical step of metastasis is migration [36]. If BMS-191011 effectively induced cell death, it may prevent migration of MDA-MB-231 cells. **Supplemental Fig.** **10** shows a typical scratch assay (or “wound heal”) imaging experiment [37]. In control experiment, there are a few live cells in the “scratched wound area” (A) at T = 0. After 4 h, live cells grew to fill the “wound” area (B). In BMS-191011 experiment, at T = 0, there are a few live cells (C). After 4 h, there were no increased number of live cells in the “wound” area (D) compared to (C). Therefore, while control (untreated) MDA-MB-231 cells can recover from the scratch within 4 h, 100 nM BMS-191011 prevented migration of MDA-MB-231 cells within 4 h. Similar results were obtained in an additional four experiments.

### BK channel opener induced arrest in cell cycle G2 phase in MDA-MB-231

**Fig. 6:**
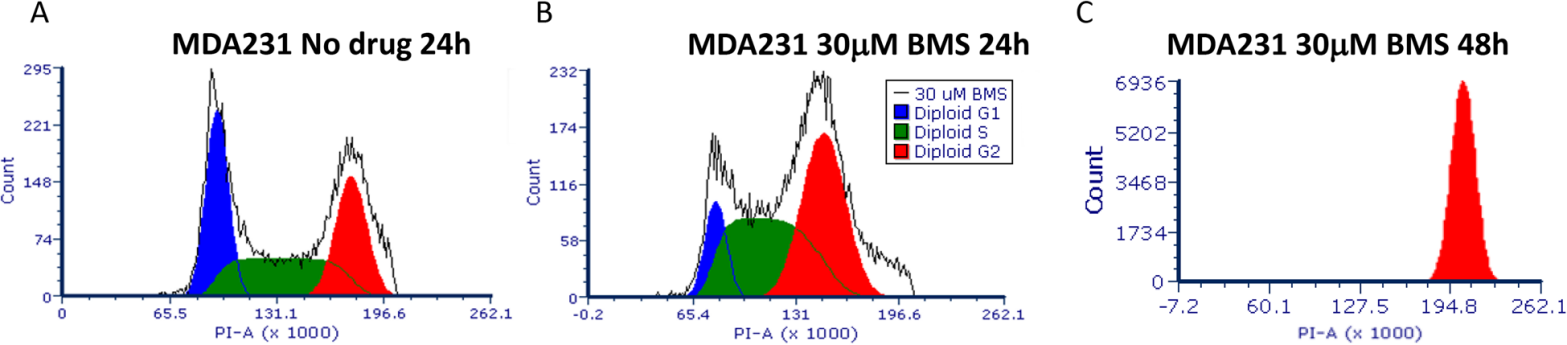
BMS-191011 induced arrest in S/G2 phases of MDA-MB-231. **a**: No treatment (control), **b**: Drug treatment for 24 h, **c**: Drug treatment for 48 h. Blue: G1 phase; Green: S phase; Red: G2 phase. Count: number of cells, PI-A: fluorescence intensity. Sub-G1 peak was gated out

Fig. 6 shows the effect of opening BK channels in MDA-MB-231 cell cycle using flow cytometry. In the absence of BMS-191011 treatment, cell distribution in G1/S/G2 phases are approximately equal (**A**), BMS-191011 treatment after 24 h resulted in arrest in S/G2 phases associated with a decrease distribution in G1 phase (**B**). After 48 h, cells are all arrested in G2 phase (**C**). As a control, we showed that DMSO (1%) had no effects on cell cycle in MDA-MB-231 cells after 24 h treatment (**Supplemental Fig.** **11**). Similar results were repeated in an additional three experiments.

### BK channel opener inhibited growth of MDA-MB-231 xenograft in NSG mice

To test the inhibitory effects of BK channel opener on TNBC tumor in vivo, we generated MDA-MB-231 xenograft in female NSG mice at 4-week old age [21]. After a sizable tumor was formed (typically after 1–2 weeks of cell injection), BMS-191011 at 100 μg/kg (or 1-2 μg/mouse) was directly injected into the tumor for better control of the dose and the potential loss of the drug due to rapid metabolism in mice. The drug was given twice a week in the treat group. For control group, saline was given twice a week. To avoid large variation in tumor sizes due to heterogeneity of breast cancer, we selected pairs of tumors (one as a control – injected PBS only, the other treated – injected drug in PBS) that had similar tumor sizes, and performed ultrasound imaging for four weeks to monitor effect of BK channel opener during the growth of tumors.

**Fig. 7:**
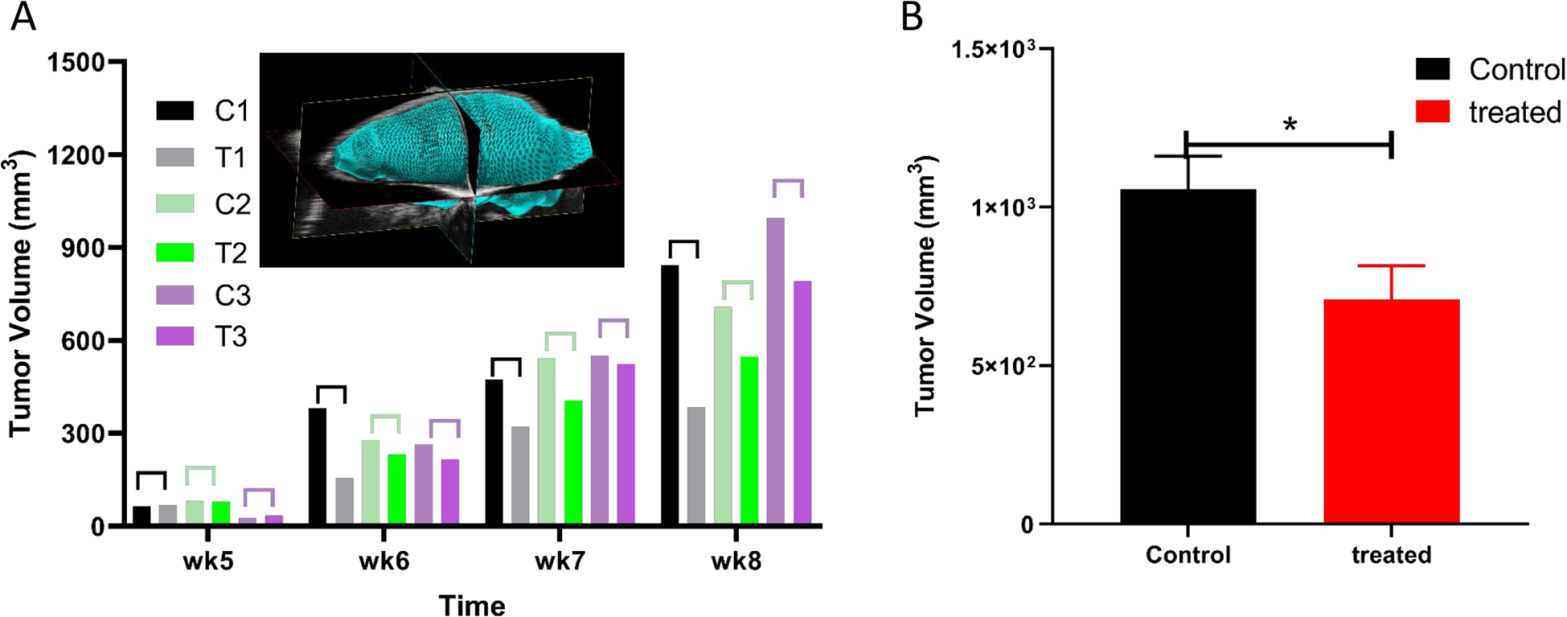
Slowing of MDA-MB-231 xenograft tumor by BK channel opener. **a**: Three paired-tumors of similar sizes (C1/T1, C2/T2, C3/T3) are shown to illustrate the inhibitory effects of BMS-191011 in tumor growth. Ultrasound imaging was performed once a week to calculate the tumor volume (mm^3^) for four weeks. A reconstruction of the tumor volume from ultrasound imaging is shown in the inset. **b**: Final average tumor volume in control (n = 8) and treated groups at week 8 (*n* = 8). *: *p* < 0.05 (t-Test was performed, two-tailed *p* = 0.0356, t = 2.32592)

Fig. 7 shows a representative example for three pairs of control and treated tumors. The injection of the drug began at week5 when three pairs (C1/T1, C2/T2, and C3/T3) had similar tumor sizes, the treated tumor (T) grew significantly slower than the control tumor (C) each week (**7A**). In week 8, averaging data showed a 33% reduction of final tumor volume in drug-treated group (T = 710 ± 105, *n* = 8) compared to the control group (C = 1056 ± 106, *n* = 8) (*p* < 0.05) (**7B**).

### BK channel opener and cardiotoxicity

Cardiac toxicity is a major concern in anti-cancer drugs [38]. **Supplemental Fig.** **12** shows the echocardiograph results for MDA-MB-231 xenograft mice treated with a high dose (0.1 mg/kg) of BMS-191011 compared to control (PBS treated) mice (*n* = 3). There are no significant differences (*p* > 0.05) between the two groups in cardiac function including heart rate, ejection fraction, left ventricular mass, and cardiac output.

Additionally, we co-cultured MDA-MB-231 with the cardiac myocytes to test the hypothesis that BK channel opener can only induce cell death in MDA-MB-231 but not in cardiac myocytes due to extremely low expression levels of KCNMA1 gene in the heart. **Supplemental Fig.** **13** shows that BMS-191011 at 20 μM indeed induced cell death only in MDA-MB-231 with little impact in cardiac myocytes after six-day incubation.

## Discussion

In the present work, we showed evidence to support a hypothesis that targeted treatment by activation of BK channels - thereby hyperpolarizing the Em - can induce cell death in TNBC while sparing healthy breast cells without cardiac toxicity. We selected a BK channel opener due to overexpression of the channels in breast cancer and large conductance of the channel. Opening large conductance potassium channels can effectively induce membrane hyperpolarization due to rapid efflux of K^+^ ions. Extremely low expression of BK channel gene expression in normal breast cells and cardiomyocytes ensures selective apoptosis in TNBC and absence of cardiac toxicity by BK channel opener.

Using microarrays, KCNMA1 gene expression was found to increase in several cancers including breast cancer [16]. Using RNA-seq data in a large breast cancer dataset in TCGA, we found overexpression of KCNMA1 in all subtypes of breast cancer. In principle, our approach of using BK opener for selective induction of cell death works for all subtypes of breast cancer. We focus on TNBC due to lack of targeted therapy in TNBC. It needs to be emphasized that mutations in KCNMA1 gene are rare – only 15 (7 in luminal A, 5 in luminal B, 2 in HER2, 1 in basal subtype) in breast cancer, although the gene is overexpressed and the cause of overexpression is unknown.

We verified that the BK channel alpha-subunit protein levels are significantly increased in MDA-MB-231 and in TNBC patient tissues compared to normal primary breast tissues. The overexpression of BK channels in breast cancer would intuitively lead to interest in inhibition. However, inhibition mandates that channels be active. This creates an apparent contradiction with the overexpression of an ion channel that if it were active should make the Em more negative, yet our research demonstrates a cell with a more positive (depolarized) resting Em. This contradiction can be resolved if the BK channels are not open at resting Em in TNBC. The lack of statistically significant correlation of relapse free survival rate with expression confirms the need to look beyond channel expression to channel function to answer this question of inhibition or activation.

We performed patch clamp studies and found that indeed < 1% of BK channels are open at -40 mV, which is near the Em in MDA-MB-231 cells [12]. This result addressed the problem of a depolarized breast cancer cell overexpressing hyperpolarizing BK channels. This result also opened a new idea that opening BK channels in breast cancer cells should hyperpolarize Em, which may halt cell growth.

We tested this concept in three TNBC cell lines, MDA-MB-231, SUM159, and HCC1143, representing different subtypes of TNBC. We used two structurally different openers (BMS-191011 and NS11021) to ensure that any effects seen in cell growth are mediated by BK channel opening. Notably, BK channel openers induced more cell death in MDA-MB-231 than in MCF-7 (a non-metastatic breast cancer cell line). One possible explanation is that BK channel protein levels are significantly higher in MDA-MB-231 than in MCF-7. Indeed, using immunofluorescence staining BK channels have been previously reported to be abundantly expressed in MDA-MB-231, very weak in MCF-7, and undetectable in MCF10A [15]. Additionally, we employed a constitutively opened mutant BK channel to demonstrate that hyperpolarization is the primary driving force to induce cell death in TNBC cells.

To explore cellular mechanisms that mediate the hyperpolarization-induced death in TNBC cells, we showed in MDA-MB-231 cells early morphology changes (shrinkage) and late activation of caspase-3 in MDA-MB-231 cells. Normotonic shrinkage of cells is a hallmark of apoptosis [34]. Caspase activation is a well-established pathway in apoptosis [39]. In addition, we showed that at low concentration (100 nM), BK channel opener was able to prevent cell migration.

During cell cycle, membrane depolarization is essential for transition of G2-phase to mitosis [9, 10]. Induced hyperpolarization during this transition can arrest cell growth by blocking DNA synthesis [9, 10]. Indeed, our results demonstrated that hyperpolarization induced by BK channel opener caused cell cycle arrest in G2 phase in MDA-MB-231.

In three size-matched tumor pairs, BMS-191011 slowed down the growth of xenograft tumors every week, indicating that opening BK channels in tumor can inhibit cell growth of TNBC cells in vivo. After four-week treatment, there was a statistical difference between the two groups, even using a small number of mice (8 per group) and twice a week drug treatment. Increasing number of mice and frequency of drug treatment may likely demonstrate a stronger statistical significance with a larger inhibitory effect in xenograft tumor growth.

A major concern in anti-cancer drugs is cardiotoxicity [38]. The KCNMA1 gene expression levels are very low in the heart (FPKM ~ 0.2 to 0.3) [40]. We performed echocardiogram on the mice to assess potential cardiac side effects of BK channel opener in cardiac functions. We found no significant changes in cardiac functions in treated mice compared to untreated mice. Furthermore, in the co-culture of TNBC cells with cardiac myocytes, we showed that BK channel opener induced TNBC cell death without significant toxicity in cardiac myocytes.

Targeted therapy has significantly increased the survival rate of breast cancer patients, while cardiotoxicity remains an increased risk for cardiovascular disease (such as left ventricular dysfunction and heart failure) in breast cancer treatment [41]. For older women (> 65 years) surviving breast cancer, the leading mortality is cardiovascular disease, not breast cancer itself [41].

Ion channels play a critical role in the hallmarks of cancer [42]. Gating of voltage-dependent ion channels is controlled by Em. Opening and closing of these channels also change Em. For example, opening of K^+^ channels causes membrane hyperpolarization (*Em becomes more negative*) due to K^+^ ions flowing out of the cell [43, 44]. On the other hand, opening of Ca^2+^ channels increases calcium influx ([Ca^2+^]_o_ ~ 1-2 mM, [Ca^2+^]_i_ ~ 100-200 nM), which contributes to membrane depolarization (*Em becomes more positive*) [45]. Calcium homeostasis is an extremely delicate process, disruption of calcium homeostasis triggers many pathological events including apoptosis [46, 47]. Many ion channels are overexpressed in cancer cells, but nearly undetectable in normal epithelial cells. For example, voltage-dependent calcium channels (VGCC) are readily detectable in breast cancer cells [48, 49], but not in human healthy mammary epithelial cells [50]. Therefore, we demonstrated that blocking VGCC can effectively inhibit growth of breast cancer cells without affecting normal breast cells [21].

Recently, voltage-gated potassium channels have attracted cancer investigators for their potential as targets in cancer therapy [51, 52]. Kv1.3 has gained particular attention due to its low expression in the heart, while overexpressed in cancer [53, 54]. We are targeting BK channel since it has the largest conductance in the potassium channel family. Opening BK channels therefore can induce the largest membrane hyperpolarization, a central strategy to breast cancer cells in which membrane potential is depolarized. BK channel expression levels in heart (RPKM ~ 0.2) is even lower than in normal breast cells (RPKM ~ 0.7), targeting BK channel activation is anticipated to have significantly less effects in cardiac functions.

## Conclusions

While KCNMA1 genes are overexpressed in all types of breast cancer, targeting it is particularly useful for TNBC due to lack of effective pharmacological therapy in TNBC patients. We propose a novel approach to inhibit TNBC growth based on depolarized Em and significantly increased BK channel gene expression in TNBC. This new strategy is independent of TNBC subtypes and yield tumor-specific destruction of TNBC without cardiac toxicity in the TNBC-cell xenograft model. While our new strategy can be clinically important for ultrasound-guided injection to the tumor for initial neoadjuvant treatment, it also provides a future research direction on development of BK channel opener that can be systemically administrated to gain significant therapeutic application.

## Supplementary information

**Additional file 1.** Supplemental Fig. 1: A BK specific antibody recognized three bands of the channel alpha subunit – the pore forming subunit. Supplemental Fig. 2: Immunohistochemistry of BK channels in TNBC patient tissues (IHC). (a) normal breast tissue, (b) TNBC tissue (BK channel expression indicated by brown color, see Methods), (c) Percentage of staining area averaged from seven TNBC and three normal breast tissues. Unpaired t-test was performed, two-tailed *p* = 0.0042, t = 3.95. Supplemental Fig. 3: BMS-191011 on human breast cancer cell lines. Upper panels show the bright-field images, lower panels show the dead cells labelled by EthD-1 dye (red). BMS-191011 (20 μM) treatment of MCF10A (left), MDA-MB-231 (middle), and MCF7 (right) after 2 days. Scale bar: 20 μm. Supplemental Fig. 4: Concentration-dependent effects of NS11021 on MDA-MB-231 after 5 days. Upper panels show the effects of NS11021 in bright field, lower panels show the dead cells labelled by EthD-1 dye (red). Scale bar: 20 μm. Supplemental Fig. 5: Concentration-dependent effects of BMS-191011 on SUM159 after 5 days. Upper panels show the bright-field images, lower panels show the dead cells labelled by EthD-1 dye (red). Scale bar: 20 μm. Supplemental Fig. 6: BMS-191011 on HCC1143. Upper panels show the effects of BMS-191011, lower panels show the dead cells labelled by EthD-1 dye (red). Left: control; Right: one day after 10 μM BMS-191011 treatment. Scale bar: 20 μm. Supplemental Fig. 7: DMSO on HCC1143. DMSO (5 μl, corresponding to the volume used for 50 μM BMS-191011) on HCC1143. Upper: after 48 h; Lower: after 96 h. Left: light image, Right: dead cells labelled by EthD-1 dye (red). Scale bar: 20 μm. Supplemental Fig. 8: MDA-MB-231 cell death induced by a constitutively open BK channel mutant, A313D. WT: wild-type BK channel, A313D: mutant channel, GFP: GFP plasmid, *: *p* < 0.05. Supplemental Fig. 9: Time-lapse imaging of early phase of apoptosis induced by BMS-191011. Three images corresponding to starting time point (control, t = 0) (A), 20 min (B), and 60 min (C) after BMS-191011 (1 μM) application are shown. Red arrows illustrate the representative cells undergoing morphological changes during the time course of BMS-191011. Supplemental Fig. 10: Prevention of MDA-MB-231 cell migration by BMS-191011. In the absence of BK channel opener (Con) (A), cells migrated to fill the gap (“wound”) after 4 h (B). Low concentration (100 nM) of BK-191011 (C) prevented the “heal” (cells filling the gap) after 4 h (D). Scratch is defined by the space within two red lines. Curved red line indicates the marker (shadowed area) used to identify the location of the scratch. Scale bar: 20 μm. Supplemental Fig. 11: DMSO does not affect cell cycle in MDA-MB-231. a: no DMSO treatment, b: DMSO treatment after 24 h. Blue: G1 phase; Green: S phase; Red: G2 phase. Count: amount of cells, PI-A: fluorescence intensity. Supplemental Fig. 12: BK channel opener does not alter cardiac functions in NSG xenograft model. The Y axis label is described in the figure. Beats per minute (BPM) is for heart rate, % for ejection fraction, mg for left ventricular mass, and mL/min for cardiac output. Results of three control and four treated mice were compared. C: control, T: BMS-191011 treated. HR: heart rate (beat per minute, BMP), EF: ejection fraction (%), LV: corrected left ventricular mass (mg), CO: cardiac output (mL/min). Unpaired t-Test was performed. For HR, *p* = 0.6621, t = 0.4595; For EF, *p* = 0.5281, t = 0.6695; For LV, *p* = 0.5209, t = 0.6816; For CO, *p* = 0.9443, t = 0.07285. Supplemental Fig. 13: BK channel opener induced cell death in MDA-MB-231, but not in cardiac myocytes. A: MDA-MB-231 stable cell line with DsRed inserted. B: H9c2 cardiac myocytes. C: co-culture of MDA-MB-231/DsRed cells (red) with H9c2 cardiac myocytes (gray) after one-day treatment of BMS-191011 (20 μM). D: co-culture of MDA-MB-231/DsRed cells (red) with H9c2 cardiac myocytes (gray) after six-day treatment of BMS-191011 (20 μM). Scale bar: 30 μm. Supplementary Fig. 15: Full WB gel blot for cropped blot Fig. 2a and b in the manuscript. Left: BK channel protein expression in MDA231, normal breast tissue, TNBC, and MB tissues. Right: beta-actin controls in in MDA231, normal breast tissue, TNBC, and MB tissues. Blots were imaged using a Licor Odyssey CLx and image studio software. Supplement Fig. 16: Full WB for Fig. 2d. Left: BK protein expression in MDA231, SUM159, MCF10A, and HCC1143. Right: beta actin controls in MDA231, SUM159, MCF10A, and HCC1143. Images were taken and processed using a Licor Osyssey CLx and image studio software. Supplemental Fig. 17: full WB for Fig. 5c. Left: caspase-3 protein expression in MDA231, HCC1143, and SUM159. Right: beta-actin protein expression in MDA231, HCC1143, and SUM159. Images were taken and processed using a Licor Osyssey CLx and image studio software. Supplemental Fig. 18: full WB for Fig. 5d. Left: total and cleaved caspase-3 protein expression in untreated MDA231, HCC1143, and SUM159. Right: total and cleaved caspase-3 protein expression in BMS-treated MDA231, BMS-treated tumor, and untreated tumor. Images were taken and processed using a Licor Osyssey CLx and image studio software.

## Data Availability

All data generated or analyzed in this study are included in this published article [and its supplementary information files].

## Abbreviations

BK: Big K, Maxi-K, Slo1, or KCa1.1
FFPE: Formalin-fixed paraffin-embedded
HER2: Human epidermal growth factor receptor 2
NSG: *NOD scid gamma*
PR: Progesterone receptor
TCGA: The Cancer Genomic Atlas
TNBC: Triple-negative breast cancer
