# Supplementary material for: Opening large-conductance potassium channels selectively induced cell death of triple-negative breast cancer
Source: BMC Cancer. 2020 Jun 26;20:595. doi: 10.1186/s12885-020-07071-1 (PMC7318490; doi:10.1186/s12885-020-07071-1)
Supplement: Supplementary file 1 — Additional file 1. Supplemental Fig. 1: A BK specific antibody recognized three bands of the channel alpha subunit – the pore forming subunit. Supplemental Fig. 2: Immunohistochemistry of BK channels in TNBC patient tissues (IHC). (a) normal breast tissue, (b) TNBC tissue (BK channel expression indicated by brown color, see Methods), (c) Percentage of staining area averaged from seven TNBC and three normal breast tissues. Unpaired t-test was performed, two-tailed p = 0.0042, t = 3.95. Supplemental Fig. 3: BMS-191011 on human breast cancer cell lines. Upper panels show the bright-field images, lower panels show the dead cells labelled by EthD-1 dye (red). BMS-191011 (20 μM) treatment of MCF10A (left), MDA-MB-231 (middle), and MCF7 (right) after 2 days. Scale bar: 20 μm. Supplemental Fig. 4: Concentration-dependent effects of NS11021 on MDA-MB-231 after 5 days. Upper panels show the effects of NS11021 in bright field, lower panels show the dead cells labelled by EthD-1 dye (red). Scale bar: 20 μm. Supplemental Fig. 5: Concentration-dependent effects of BMS-191011 on SUM159 after 5 days. Upper panels show the bright-field images, lower panels show the dead cells labelled by EthD-1 dye (red). Scale bar: 20 μm. Supplemental Fig. 6: BMS-191011 on HCC1143. Upper panels show the effects of BMS-191011, lower panels show the dead cells labelled by EthD-1 dye (red). Left: control; Right: one day after 10 μM BMS-191011 treatment. Scale bar: 20 μm. Supplemental Fig. 7: DMSO on HCC1143. DMSO (5 μl, corresponding to the volume used for 50 μM BMS-191011) on HCC1143. Upper: after 48 h; Lower: after 96 h. Left: light image, Right: dead cells labelled by EthD-1 dye (red). Scale bar: 20 μm. Supplemental Fig. 8: MDA-MB-231 cell death induced by a constitutively open BK channel mutant, A313D. WT: wild-type BK channel, A313D: mutant channel, GFP: GFP plasmid, *: p < 0.05. Supplemental Fig. 9: Time-lapse imaging of early phase of apoptosis induced by BMS-191011. Three images corresponding [file 12885_2020_7071_MOESM1_ESM.docx]

**Supplementary information**

**Title**

Opening Large-Conductance Potassium Channels Selectively Induced Cell Death of Triple-Negative Breast Cancer

**Authors**

Gina Sizemore ^1^, Sarah McLaughlin ^2^, Mackenzie Newman ^3^, Kathleen Brundage ^4^, Amanda Ammer ^2^, Karen Martin ^2^, Elena Pugacheva ^5^, James Coad ^6^, Malcolm D. Mattes ^7^, Han-Gang Yu ^3,*^

**Affiliations**

^1^ Clinical and Translational Sciences Institute, West Virginia University

^2^ Animal Models & Imaging Facility, Cancer Institute, West Virginia University

^3^ Department of Physiology & Pharmacology, West Virginia University

^4^ Department of Microbiology and Cell Biology, Flow Cytometry Facility, West Virginia University

^5^ Department of Biochemistry, Cancer Institute, West Virginia University

^6^ Department of Pathology, West Virginia University

^7^ Department of Radiation Oncology, Cancer Institute, West Virginia University

**Corresponding author**

Han-Gang Yu, PhD

Department of Physiology and Pharmacology

West Virginia University

Morgantown, WV 26506

Phone: 304-293-2324

Email: [hyu@hsc.wvu.edu](mailto:hyu@hsc.wvu.edu)

**Supplemental Figures and Legends**

**
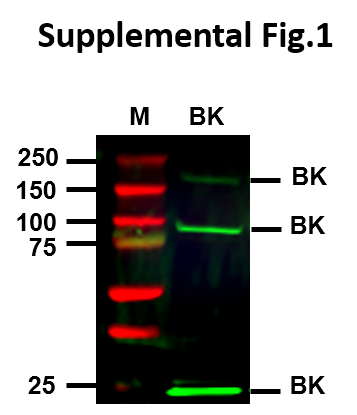
**

**Supplemental Figure 1: A BK specific antibody recognized three bands of the channel alpha subunit – the pore forming subunit.**

**
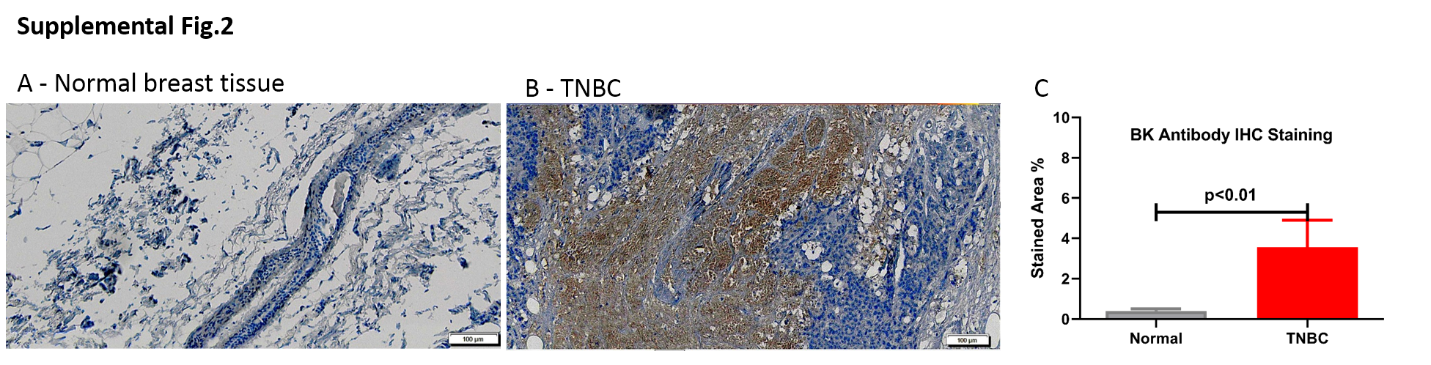
**

**Supplemental Figure 2: Immunohistochemistry of BK channels in TNBC patient tissues (IHC).** (a) normal breast tissue, (b) TNBC tissue (BK channel expression indicated by brown color, see Methods), (c) Percentage of staining area averaged from seven TNBC and three normal breast tissues. Unpaired t-test was performed, two-tailed p=0.0042, t=3.95.

**
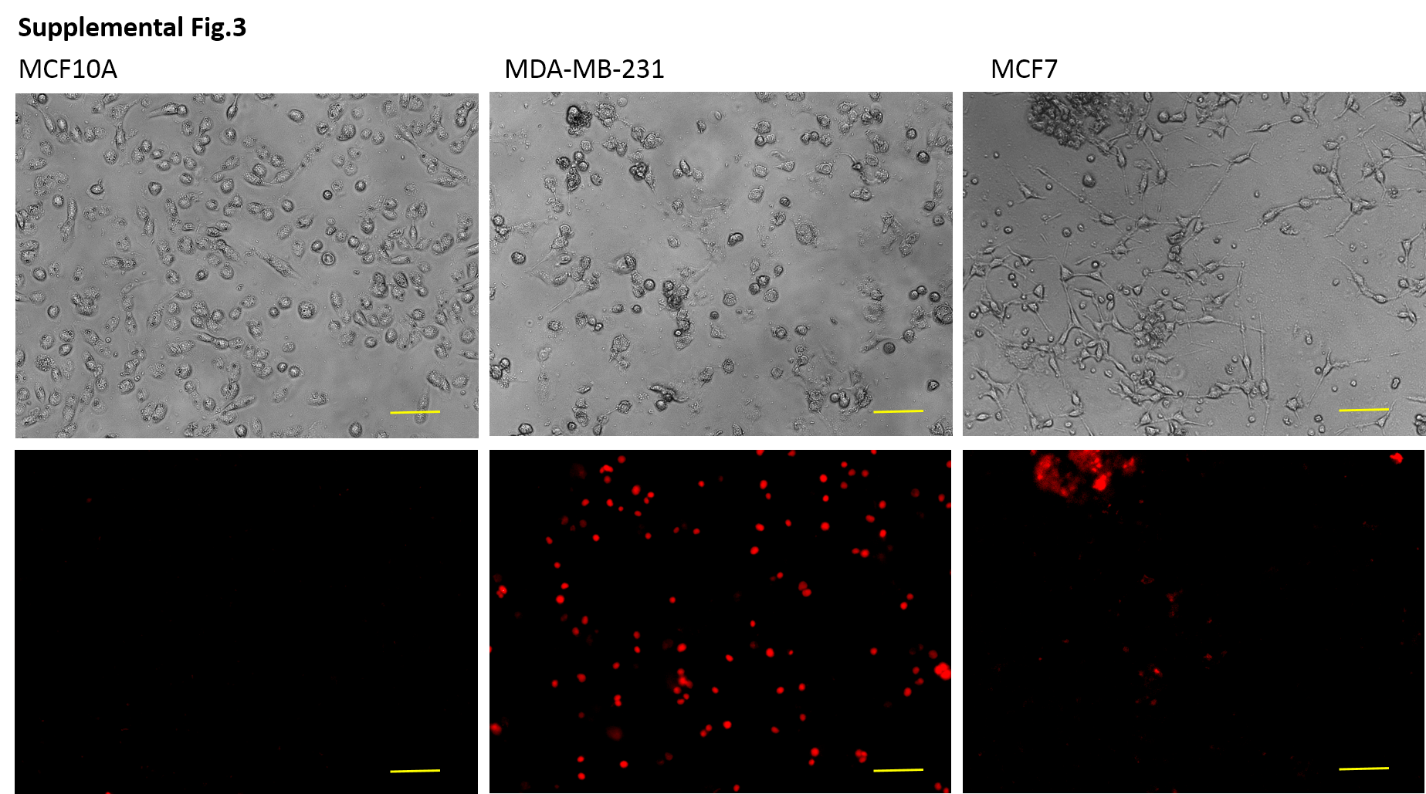
**

**Supplemental Figure 3: BMS-191011 on human breast cancer cell lines.** Upper panels show the bright-field images, lower panels show the dead cells labelled by EthD-1 dye (red). BMS-191011 (20μM) treatment of MCF10A (left), MDA-MB-231 (middle), and MCF7 (right) after 2 days. Scale bar: 20μm.


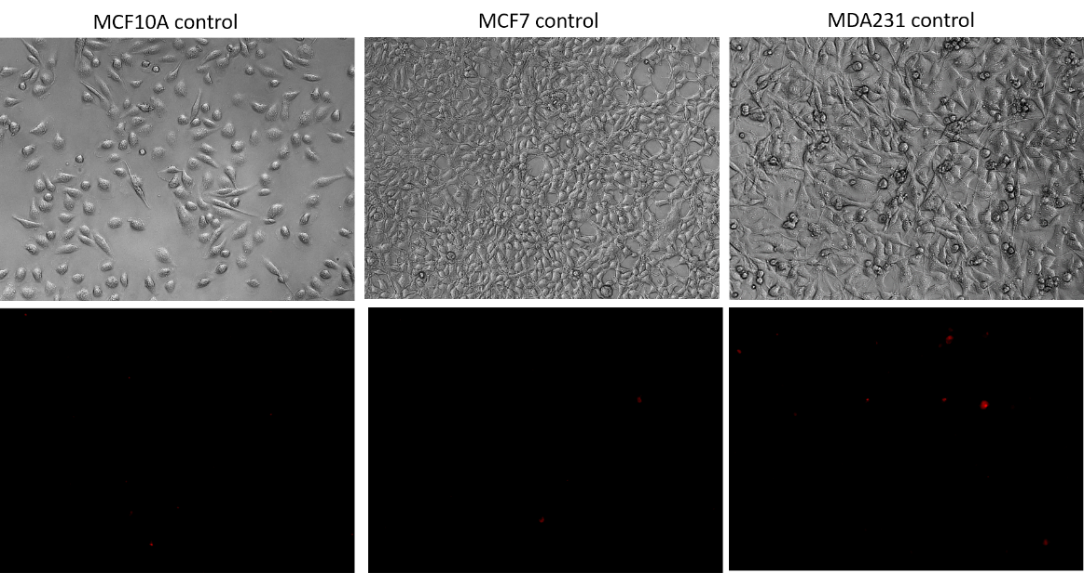
Controls for SFig3: day 0, bright field (upper) and fluorescent data (lower). Red dots indicate dead cells.


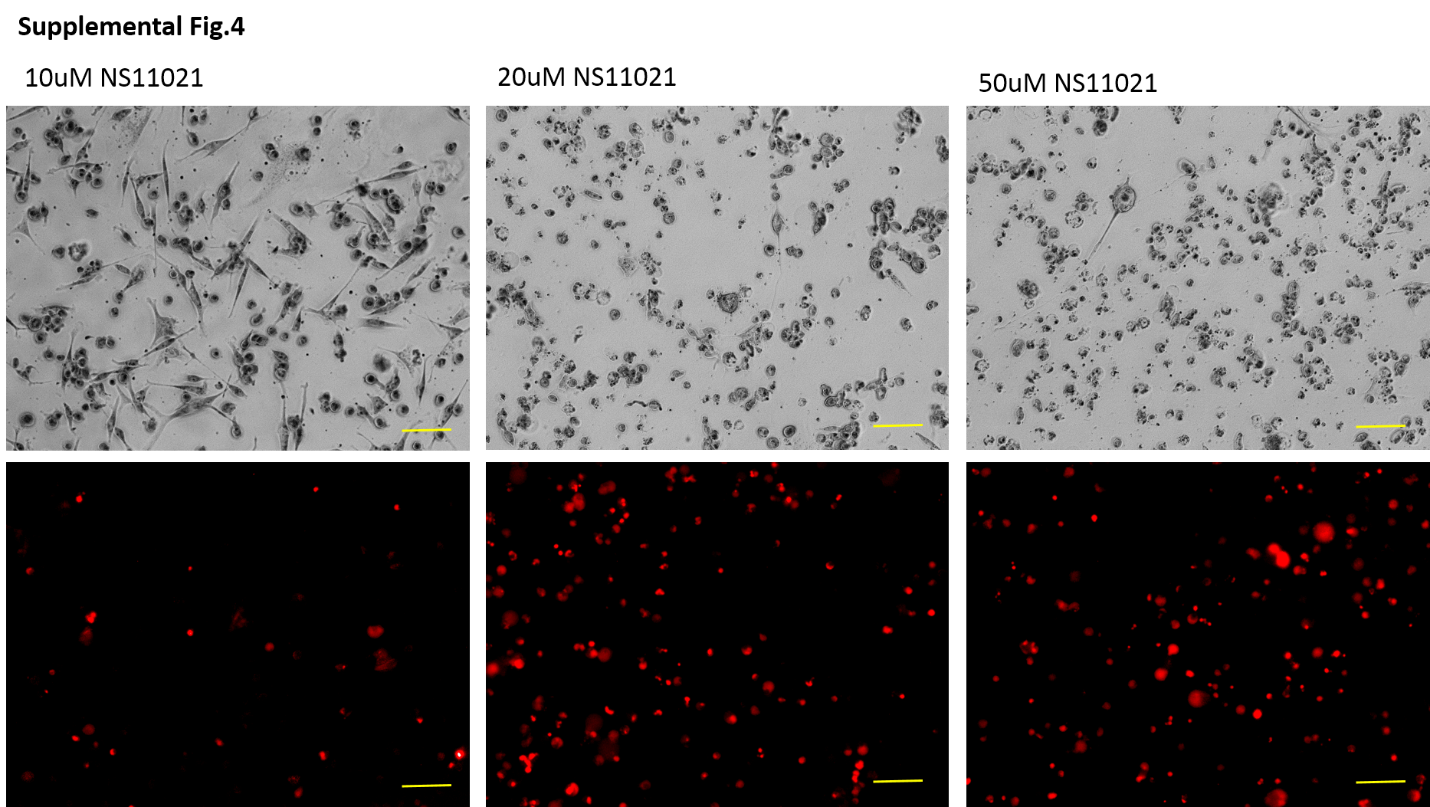


**Supplemental Figure 4: Concentration-dependent effects of NS11021 on MDA-MB-231 after 5 days.** Upper panels show the effects of NS11021 in bright field, lower panels show the dead cells labelled by EthD-1 dye (red). Scale bar: 20μm.


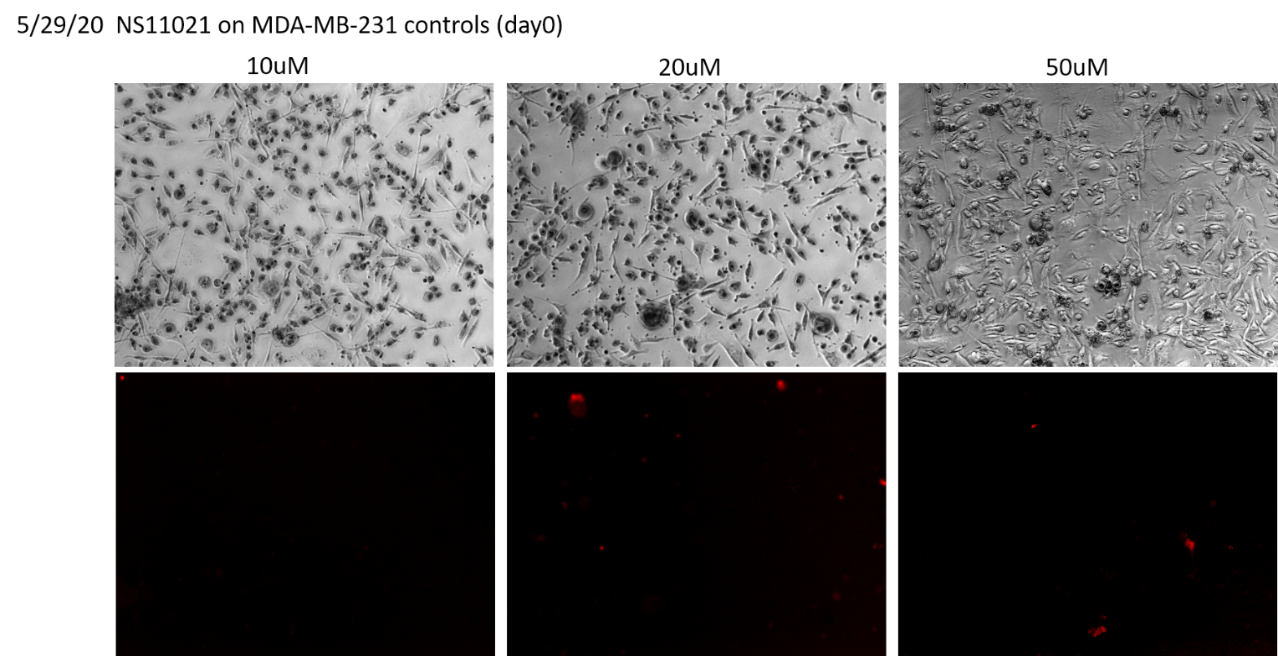
Controls for SFig4: day 0, bright field (upper) and fluorescent data (lower). Red dots indicate dead cells.

**
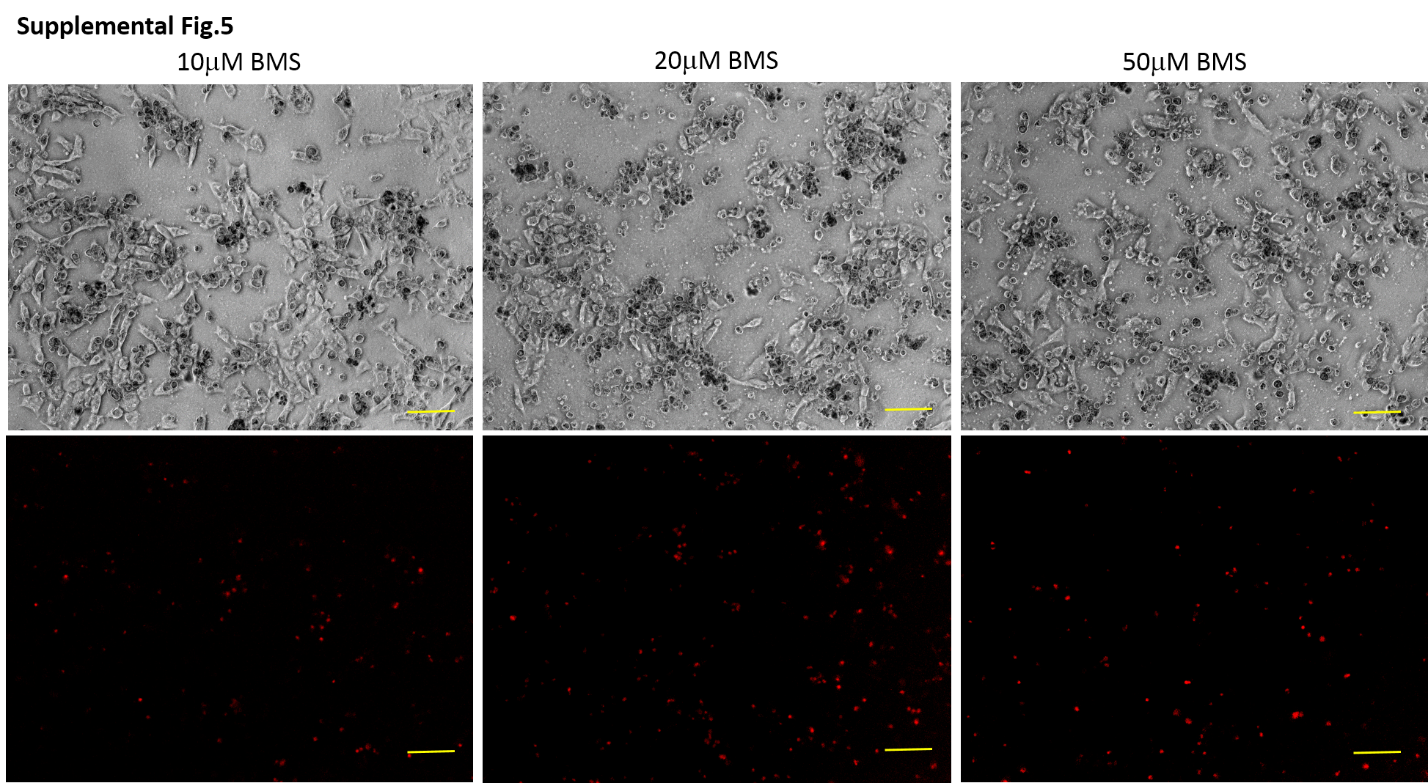
**

**Supplemental Figure 5: Concentration-dependent effects of BMS-191011 on SUM159 after 5 days.** Upper panels show the bright-field images, lower panels show the dead cells labelled by EthD-1 dye (red). Scale bar: 20μm.


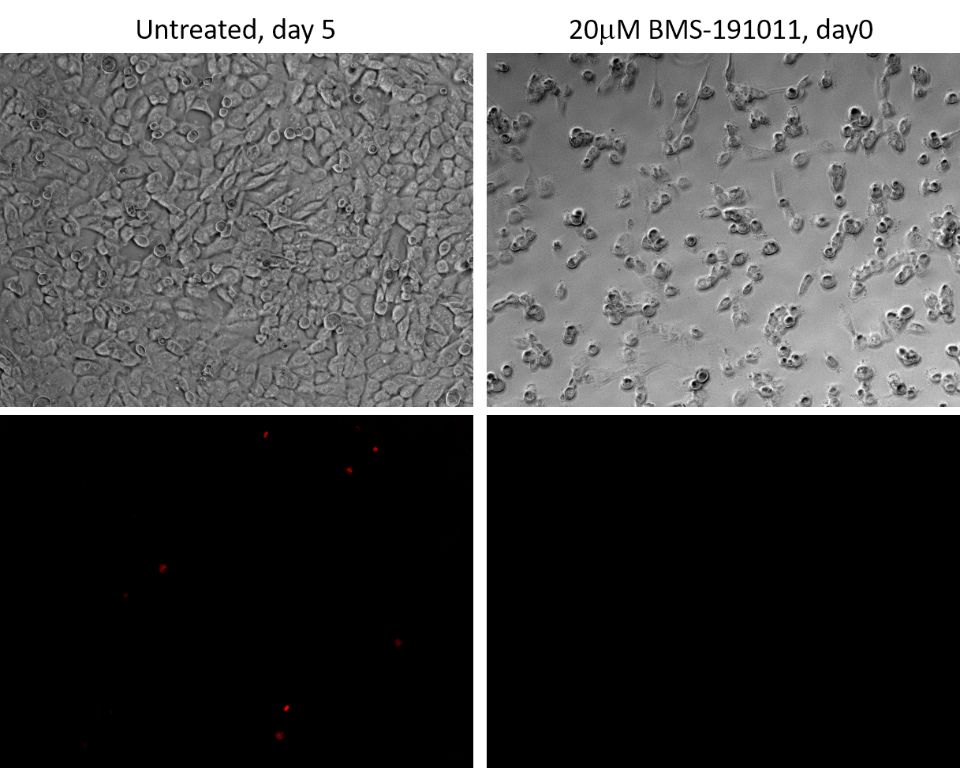
Controls for SFig5: bright field (upper) and fluorescent data (lower). Red dots indicate dead cells.


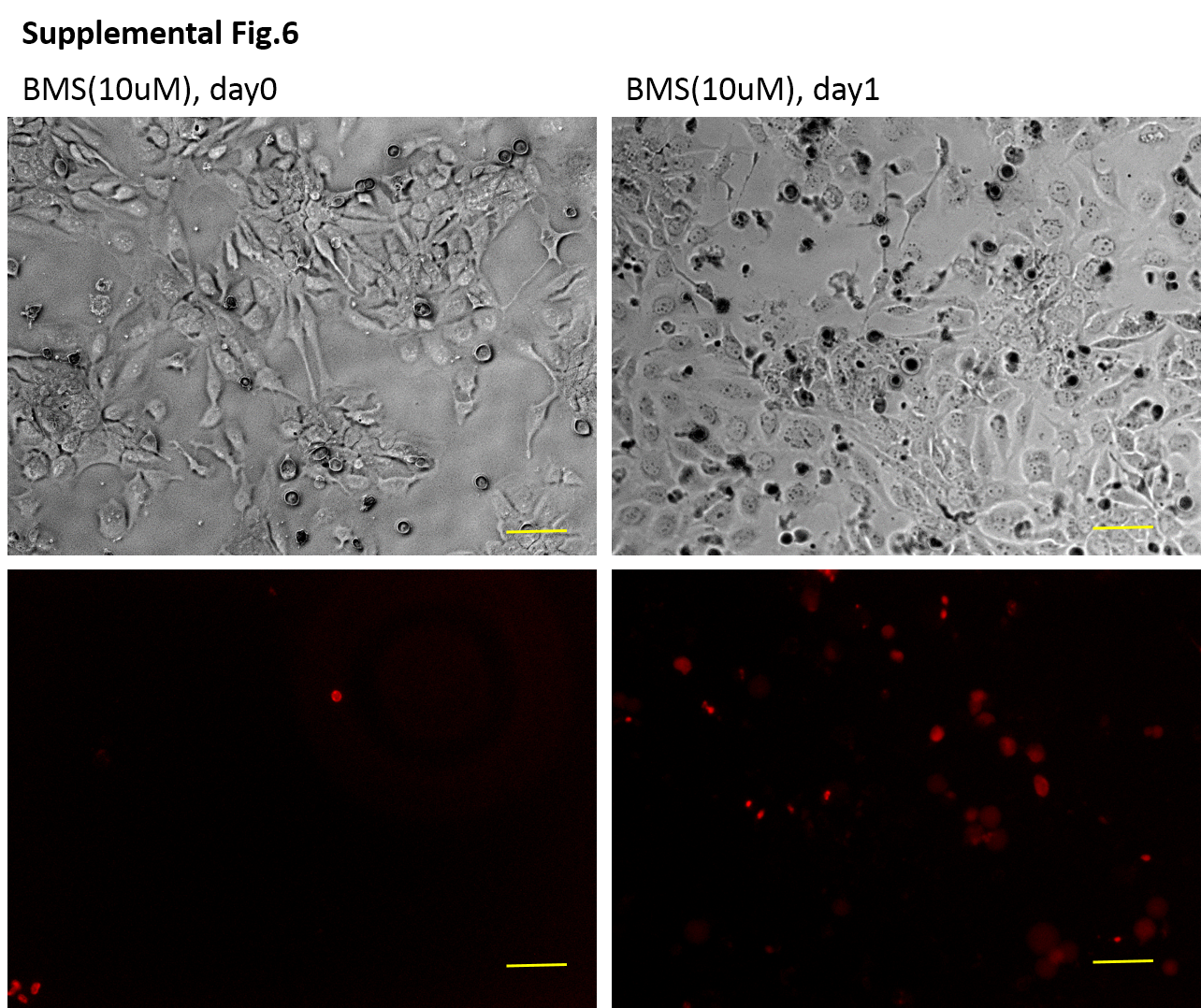


**Supplemental Figure 6: BMS-191011 on HCC1143.** Upper panels show the effects of BMS-191011, lower panels show the dead cells labelled by EthD-1 dye (red). Left: control; Right: one day after 10μM BMS-191011 treatment. Scale bar: 20μm.


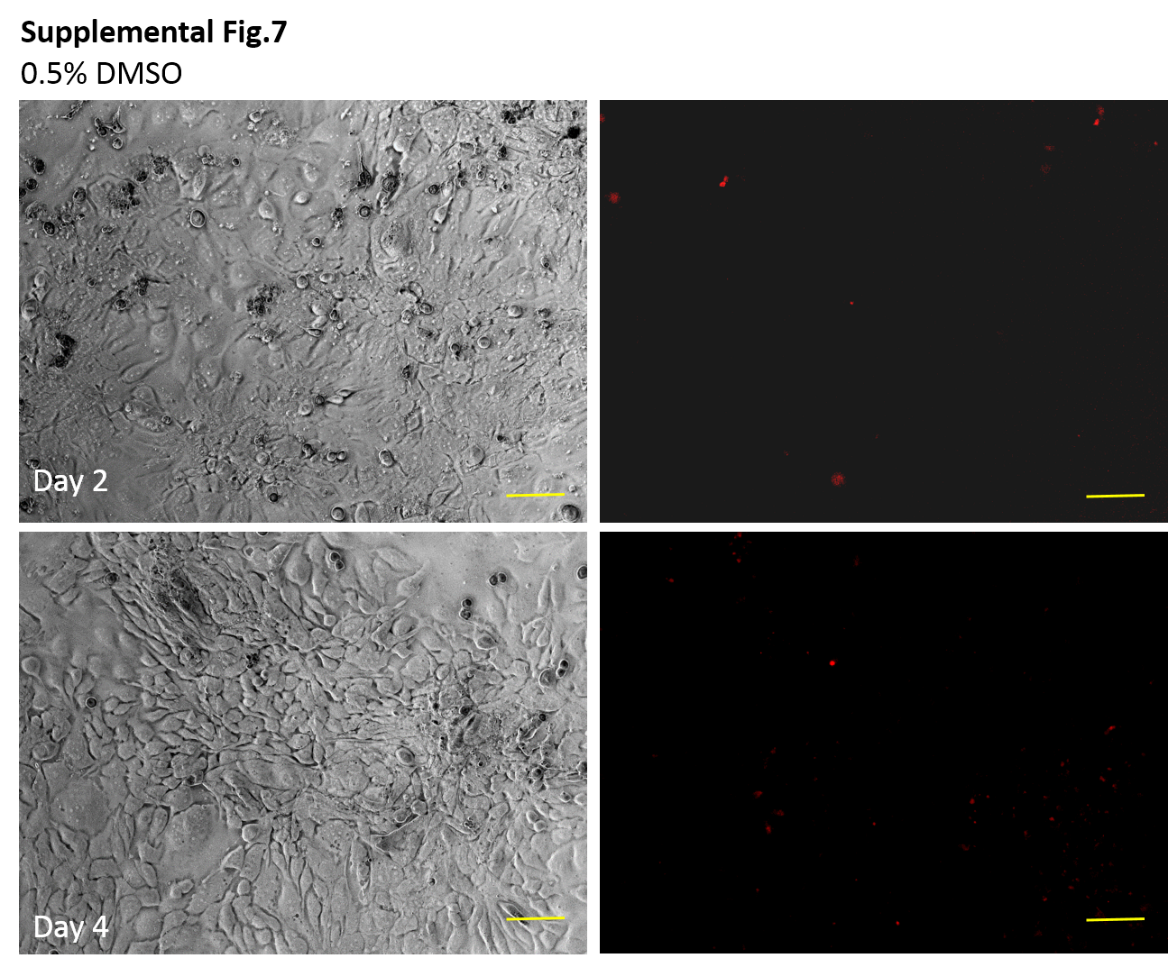


**Supplemental Figure 7: DMSO on HCC1143.** DMSO (5μl, corresponding to the volume used for 50μM BMS-191011) on HCC1143. Upper: after 48hr; Lower: after 96hr. Left: light image, Right: dead cells labelled by EthD-1 dye (red). Scale bar: 20μm.


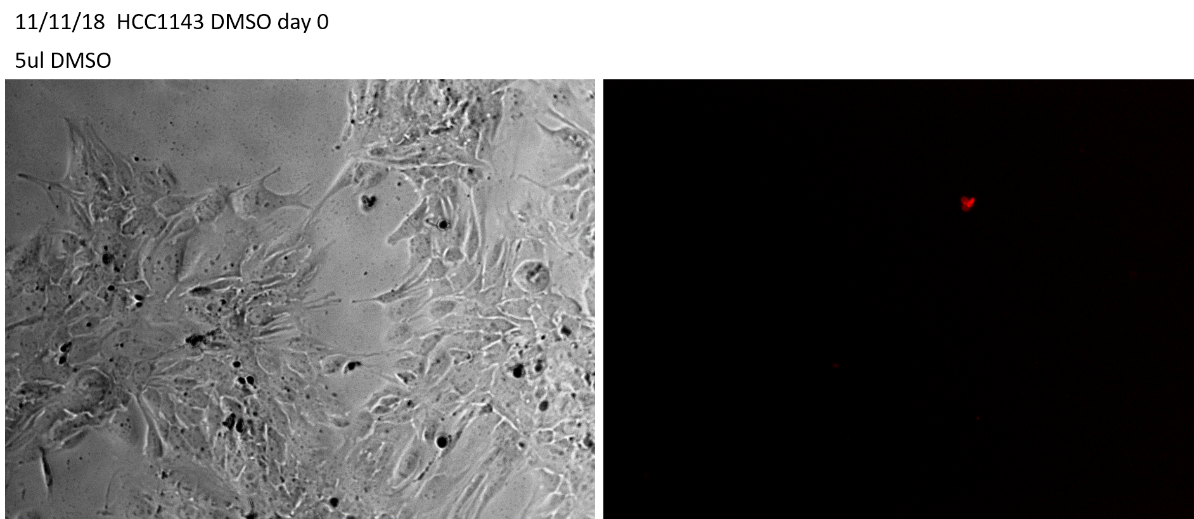
Controls for SFig7: day0. bright field (upper) and fluorescent data (lower). Red dots indicate dead cells. 5μL in 1ml medium makes 0.5%


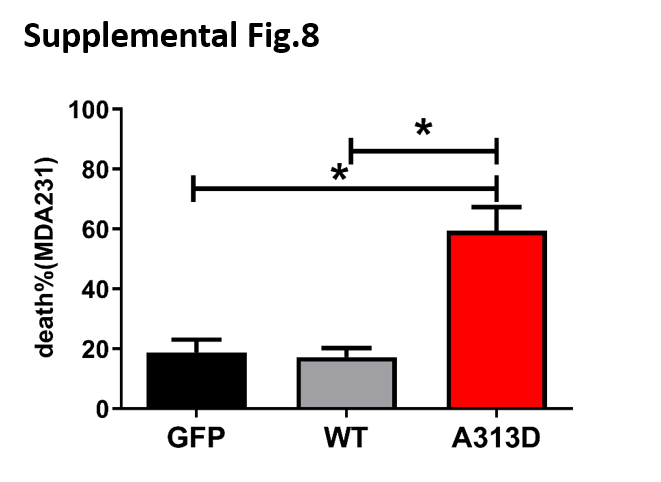


**Supplemental Figure 8: MDA-MB-231 cell death induced by a constitutively open BK channel mutant, A313D.** WT: wild-type BK channel, A313D: mutant channel, GFP: GFP plasmid, *: p<0.05.

**
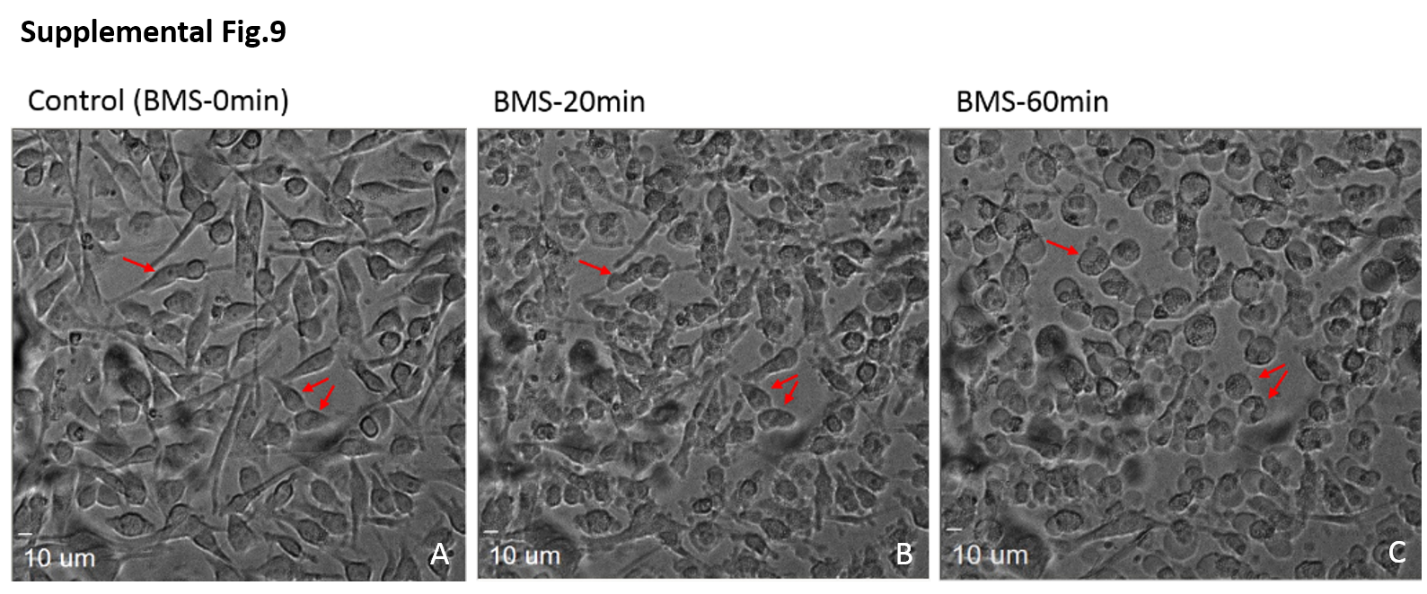
**

**Supplemental Figure 9: Time-lapse imaging of early phase of apoptosis induced by BMS-191011**. Three images corresponding to starting time point (control, t=0) (A), 20min (B), and 60min (C) after BMS-191011 (1μM) application are shown. Red arrows illustrate the representative cells undergoing morphological changes during the time course of BMS-191011.


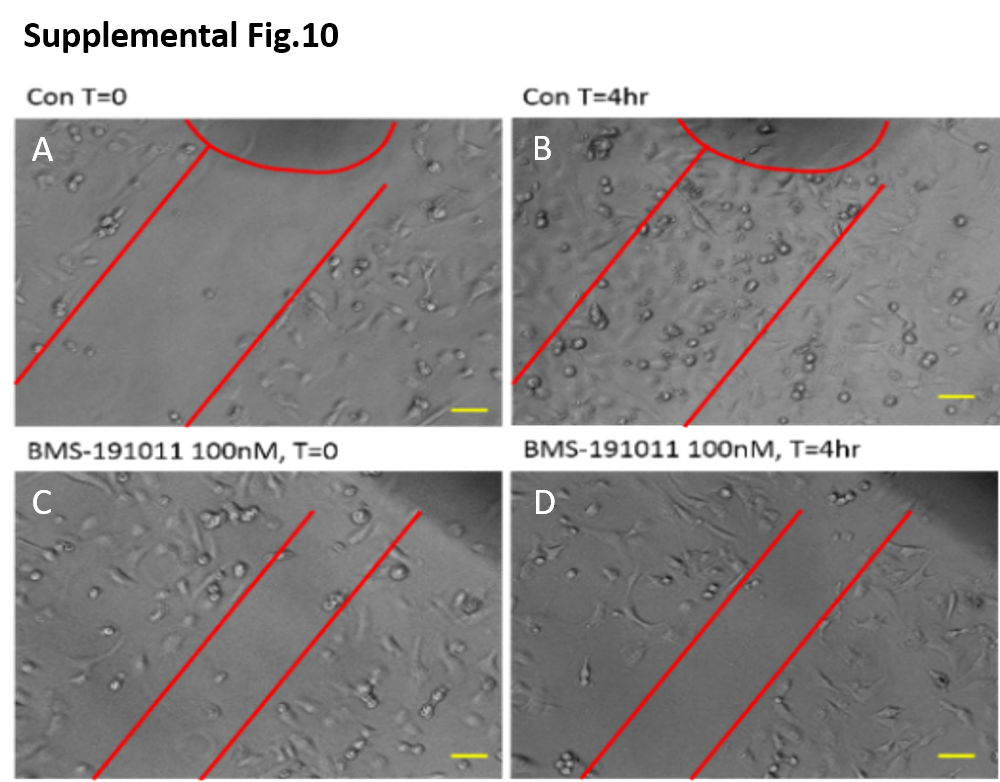


**Supplemental Figure 10: Prevention of MDA-MB-231 cell migration by BMS-191011.** In the absence of BK channel opener (Con) (A), cells migrated to fill the gap (“wound”) after 4hrs (B). Low concentration (100nM) of BK-191011 (C) prevented the “heal” (cells filling the gap) after 4hrs (D). Scratch is defined by the space within two red lines. Curved red line indicates the marker (shadowed area) used to identify the location of the scratch. Scale bar: 20μm.


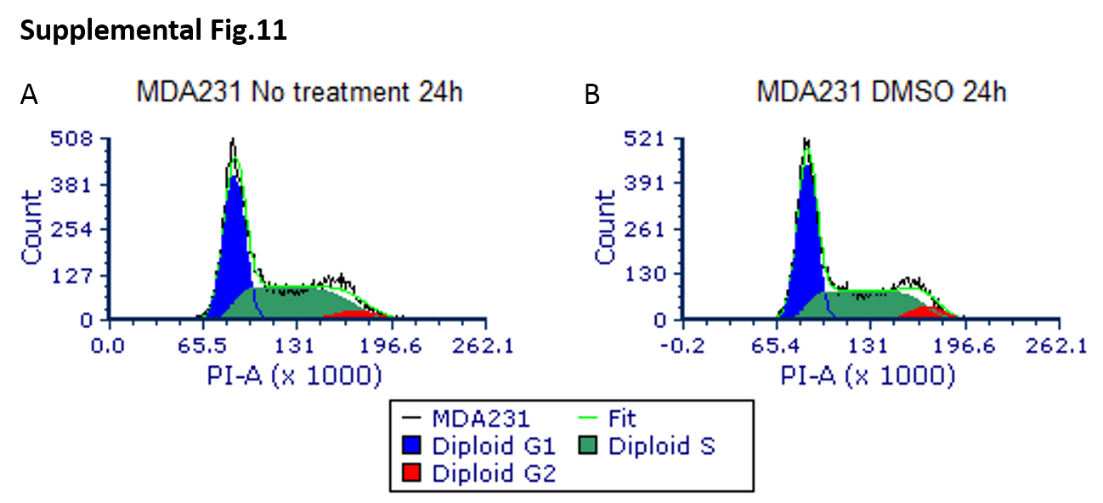


**Supplemental Figure 11: DMSO does not affect cell cycle in MDA-MB-231.** a: no DMSO treatment, b: DMSO treatment after 24h. Blue: G1 phase; Green: S phase; Red: G2 phase. Count: amount of cells, PI-A: fluorescence intensity.


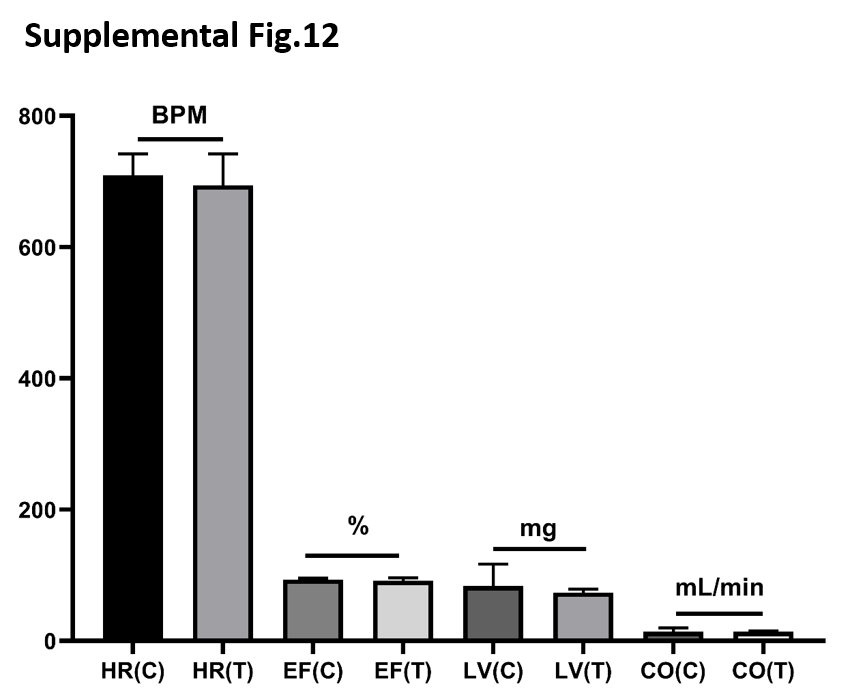


**Supplemental Figure 12: BK channel opener does not alter cardiac functions in NSG xenograft model.** The Y axis label is described in the figure. Beats per minute (BPM) is for heart rate, % for ejection fraction, mg for left ventricular mass, and mL/min for cardiac output. Results of three control and four treated mice were compared. C: control, T: BMS-191011 treated. HR: heart rate (beat per minute, BMP), EF: ejection fraction (%), LV: corrected left ventricular mass (mg), CO: cardiac output (mL/min). Unpaired t-Test was performed. For HR, p=0.6621, t=0.4595; For EF, p=0.5281, t=0.6695; For LV, p=0.5209, t=0.6816; For CO, p=0.9443, t=0.07285.


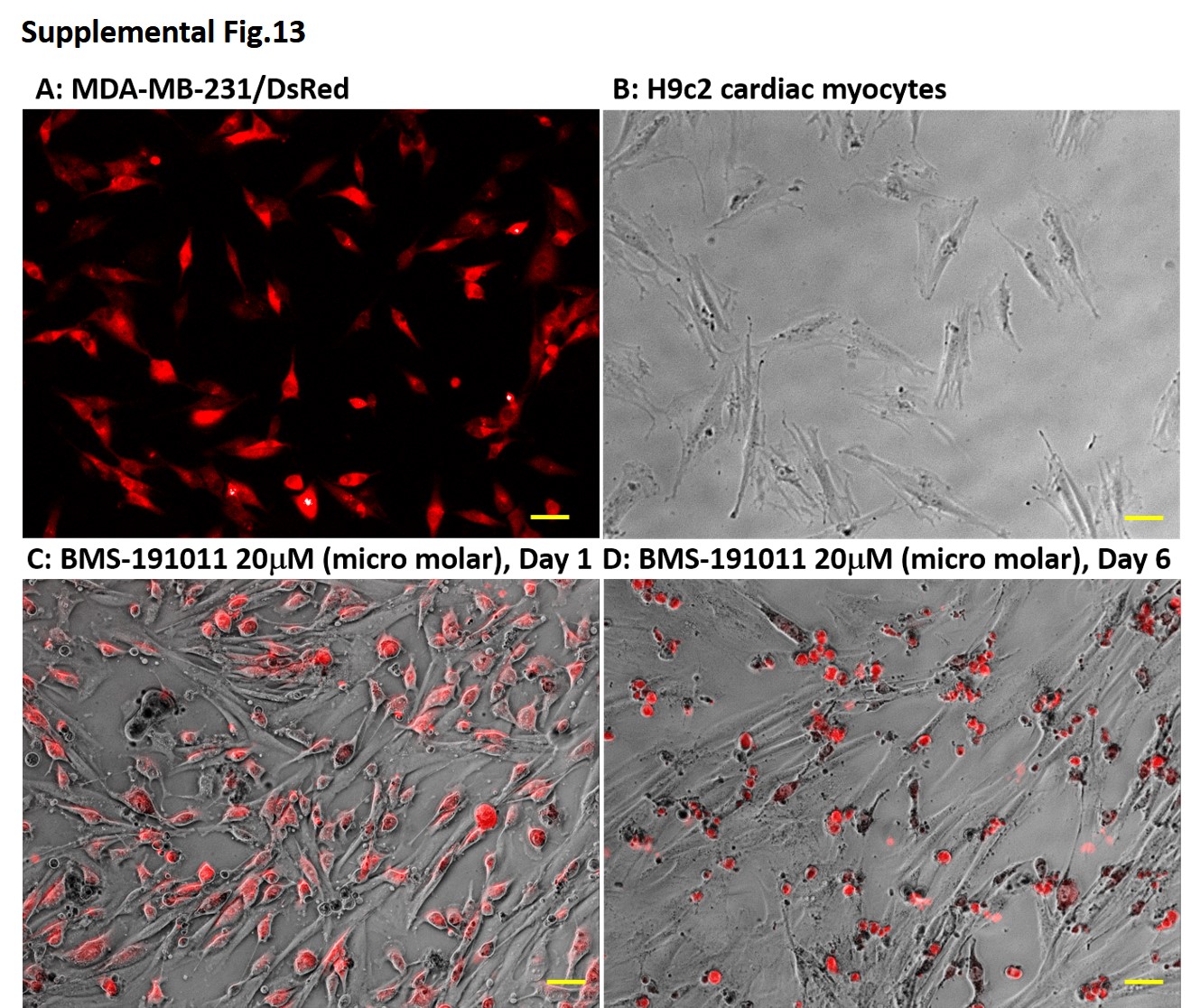


**Supplemental Figure 13: BK channel opener induced cell death in MDA-MB-231, but not in cardiac myocytes.** A: MDA-MB-231 stable cell line with DsRed inserted. B: H9c2 cardiac myocytes. C: co-culture of MDA-MB-231/DsRed cells (red) with H9c2 cardiac myocytes (gray) after one-day treatment of BMS-191011 (20μM). D: co-culture of MDA-MB-231/DsRed cells (red) with H9c2 cardiac myocytes (gray) after six-day treatment of BMS-191011 (20μM). Scale bar: 30μm.


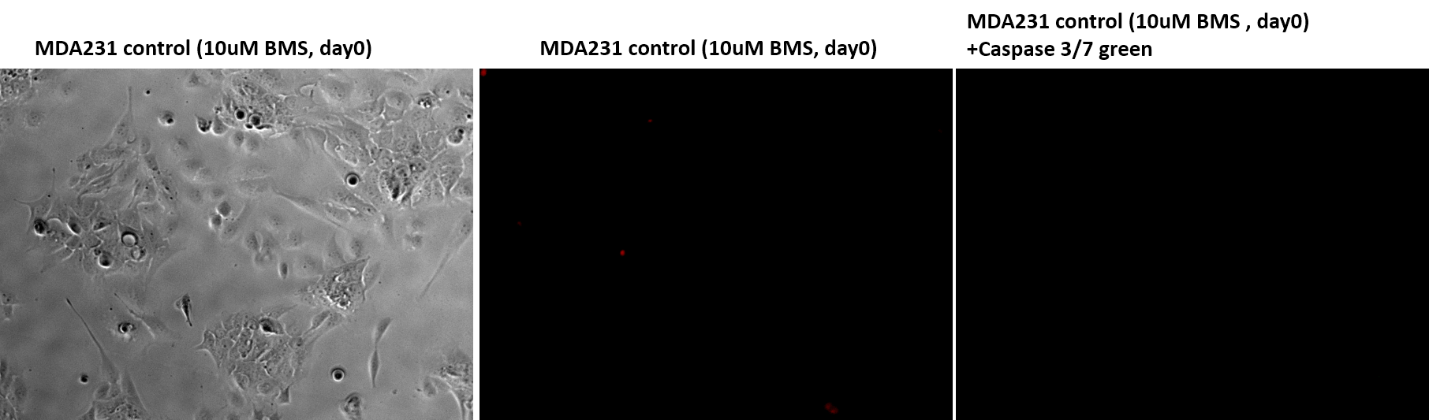


Supplemental Figure 14: control data for Figure 5. At day0, bright field, fluorescent red (ETHD-1), and fluorescent green (caspase 3/7 dye)


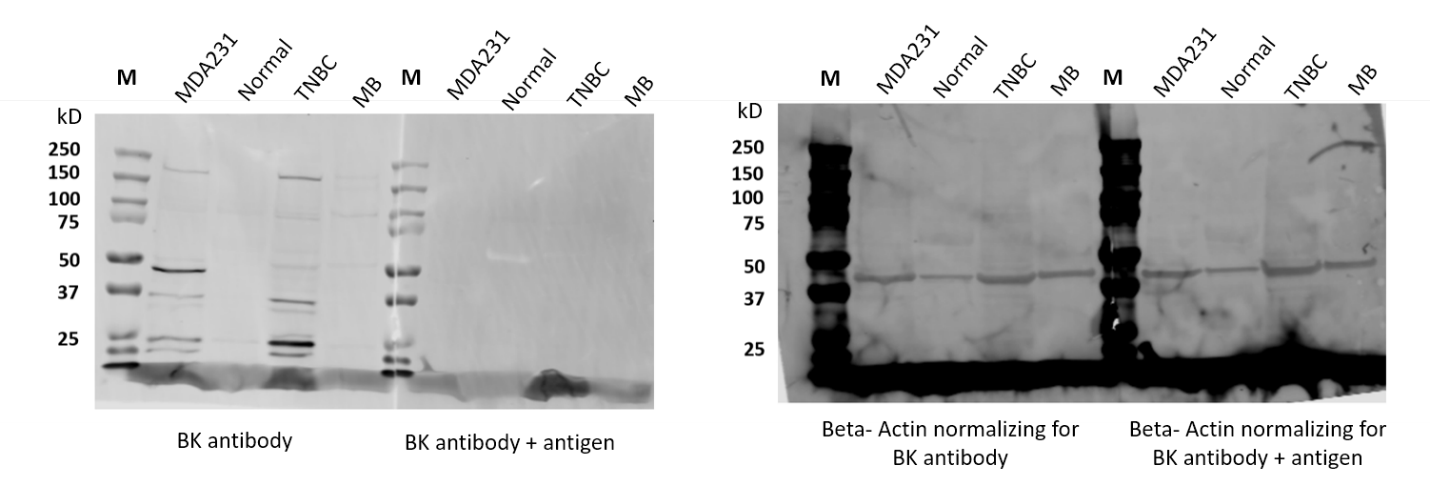


Supplementary Figure 15: Full WB gel blot for cropped blot Figure 2A and 2B in the manuscript. Left: BK channel protein expression in MDA231, normal breast tissue, TNBC, and MB tissues. Right: beta-actin controls in in MDA231, normal breast tissue, TNBC, and MB tissues. Blots were imaged using a Licor Odyssey CLx and image studio software.


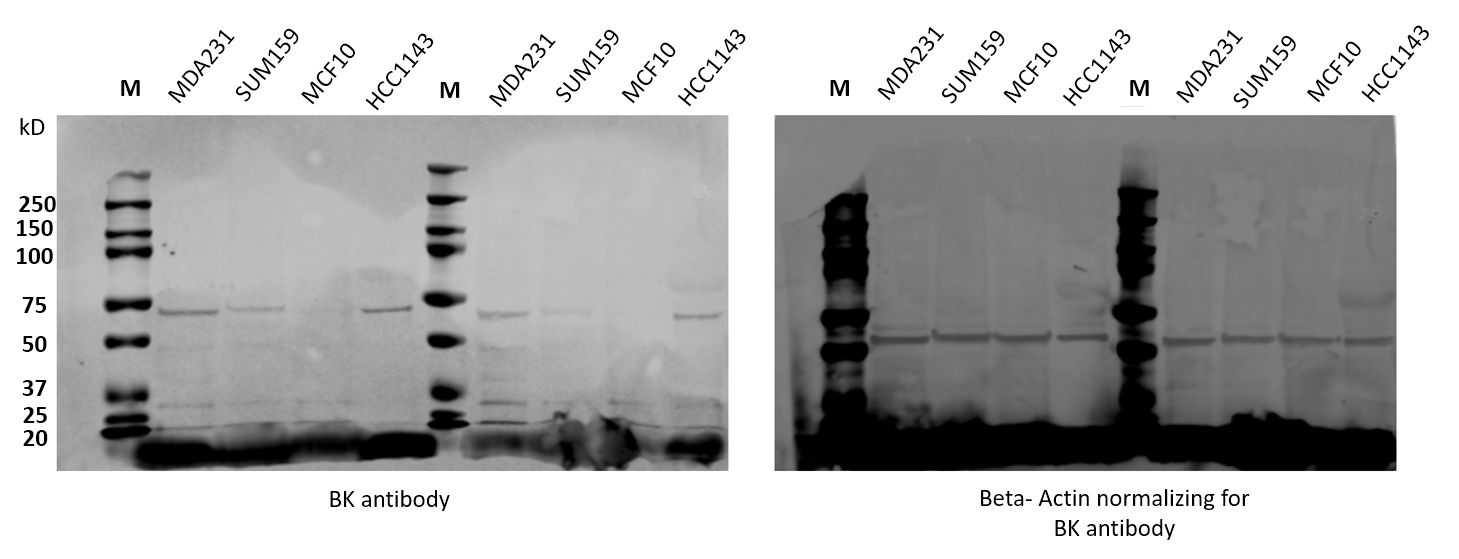


Supplement Figure 16: Full WB for Figure 2d. Left: BK protein expression in MDA231, SUM159, MCF10A, and HCC1143. Right: beta actin controls in MDA231, SUM159, MCF10A, and HCC1143. Images were taken and processed using a Licor Osyssey CLx and image studio software.


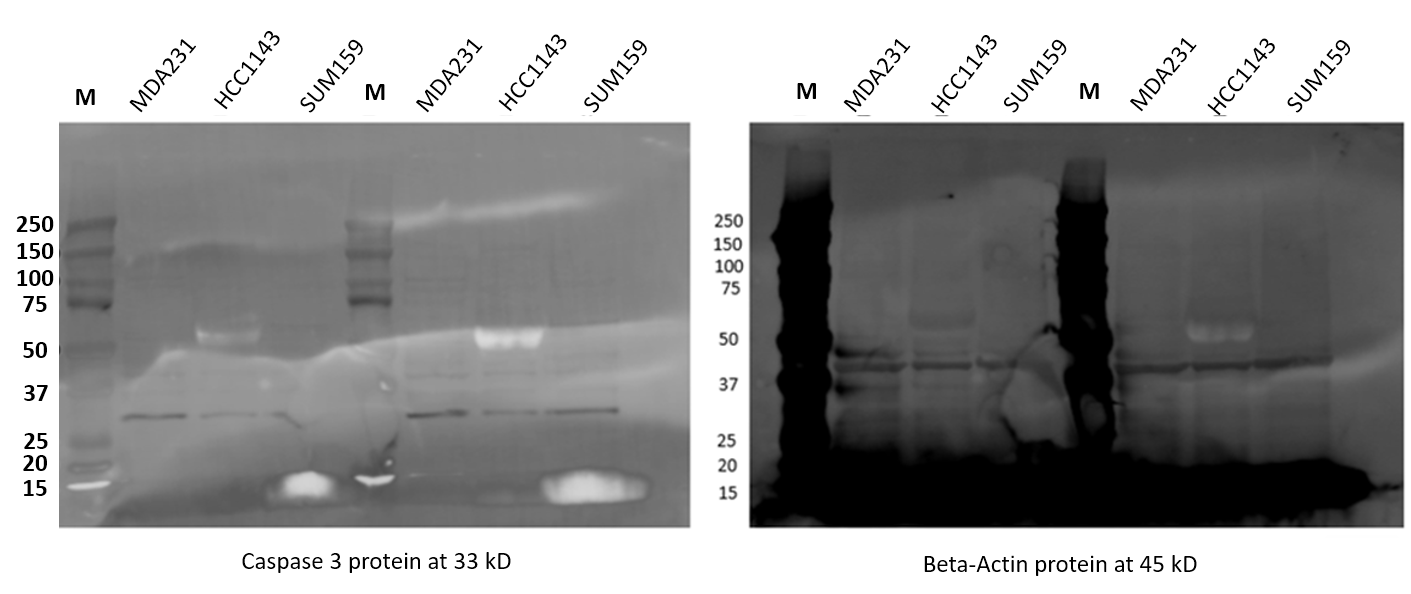


Supplemental Figure 17: full WB for Figure 5C. Left: caspase-3 protein expression in MDA231, HCC1143, and SUM159. Right: beta-actin protein expression in MDA231, HCC1143, and SUM159. Images were taken and processed using a Licor Osyssey CLx and image studio software.


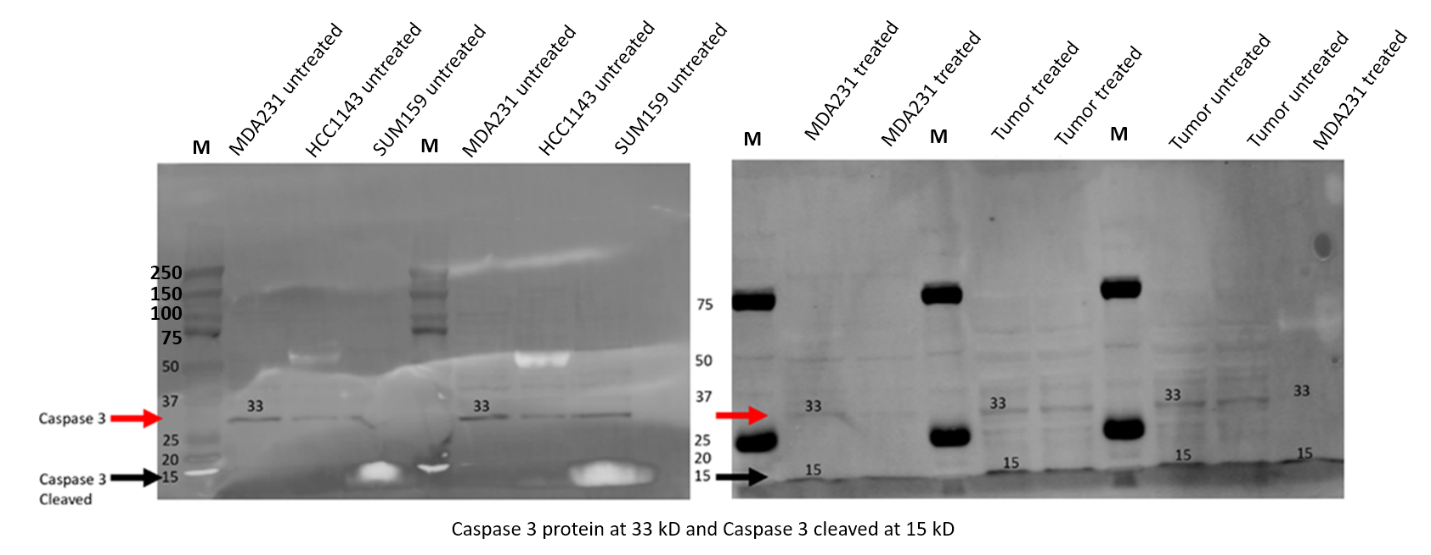


Supplemental Figure 18: full WB for Figure 5D. Left: total and cleaved caspase-3 protein expression in untreated MDA231, HCC1143, and SUM159. Right: total and cleaved caspase-3 protein expression in BMS-treated MDA231, BMS-treated tumor, and untreated tumor. Images were taken and processed using a Licor Osyssey CLx and image studio software.
